# Predicting response and toxicity to immune checkpoint inhibitors in lung cancer using antibodies to frameshift neoantigens

**DOI:** 10.1186/s12967-023-04172-w

**Published:** 2023-05-22

**Authors:** Luhui Shen, Justin R. Brown, Stephen Albert Johnston, Mehmet Altan, Kathryn F. Sykes

**Affiliations:** 1 Calviri, Inc, 850 N 5th St., Phoenix, AZ 85004 USA; 2 MD Anderson Cancer Center, Department of Thoracic-Head & Neck Medical Oncology, Division of Cancer Medicine, Houston, TX USA

**Keywords:** Checkpoint inhibitors, Immune-related adverse events, Frameshift neoantigens, Peptide microarrays, Predictive diagnostic, Lung cancer, Monotherapy, Biomarkers

## Abstract

**Purpose:**

To evaluate a new class of blood-based biomarkers, anti-frameshift peptide antibodies, for predicting both tumor responses and adverse immune events to immune checkpoint inhibitor (ICI) therapies in advanced lung cancer patients.

**Experimental design:**

Serum samples were obtained from 74 lung cancer patients prior to palliative PD-(L)1 therapies with subsequently recorded tumor responses and immune adverse events (irAEs). Pretreatment samples were assayed on microarrays of frameshift peptides (FSPs), representing ~ 375,000 variant peptides that tumor cells can be informatically predicted to produce from translated mRNA processing errors. Serum-antibodies specifically recognizing these ligands were measured. Binding activities preferentially associated with best-response and adverse-event outcomes were determined. These antibody bound FSPs were used in iterative resampling analyses to develop predictive models of tumor response and immune toxicity.

**Results:**

Lung cancer serum samples were classified based on predictive models of ICI treatment outcomes. Disease progression was predicted pretreatment with ~ 98% accuracy in the full cohort of all response categories, though ~ 30% of the samples were indeterminate. This model was built with a heterogeneous sample cohort from patients that (i) would show either clear response or stable outcomes, (ii) would be administered either single or combination therapies and (iii) were diagnosed with different lung cancer subtypes. Removing the stable disease, combination therapy or SCLC groups from model building increased the proportion of samples classified while performance remained high. Informatic analyses showed that several of the FSPs in the all-response model mapped to translations of variant mRNAs from the same genes. In the predictive model for treatment toxicities, binding to irAE-associated FSPs provided 90% accuracy pretreatment, with no indeterminates. Several of the classifying FSPs displayed sequence similarity to self-proteins.

**Conclusions:**

Anti-FSP antibodies may serve as biomarkers for predicting ICI outcomes when tested against ligands corresponding to mRNA-error derived FSPs. Model performances suggest this approach might provide a single test to predict treatment response to ICI and identify patients at high risk for immunotherapy toxicities.

**Supplementary Information:**

The online version contains supplementary material available at 10.1186/s12967-023-04172-w.

## Introduction

Immune checkpoint inhibitors (ICIs) are arguably the most singular advancement in cancer treatment since chemotherapy became the standard of care many decades ago. Currently the most frequently used immunotherapies target the PD-(L)1 pathway [1]. Over expression of this ligand on many tumors enables inhibitory immune signaling in the tumor microenvironment (TME). ICIs have been widely used for non-small cell lung cancer (NSCLC) following the demonstration of significant anti-tumor activity with anti-PD-(L)1 therapy in metastatic patients [2–6]. Lung cancer is the leading causes of cancer deaths world-wide [7]; NSCLC represents 85% of this population [8]. Several anti-PD-(L)1 agents administered as monotherapies or in combination regimens have received FDA approval [9, 10]. They are now first-line therapies for lung and several other cancers [11]. Nonetheless, current enthusiasm is tempered by low patient response rates [12, 13]. Concomitantly, costs are high and immune-related adverse effects (irAEs) can occur. These side effects arise from aberrant activation of autoreactive T cells and can affect nearly every organ, increasing the risk of therapy-related mortality and morbidity. Their onset and duration are unpredictable and predisposing factors for developing them remain unclear [14, 15].

For all cancer patients, the level of PD-L1 protein expression on the surface of biopsied tumor cells, assessed by immunohistochemistry (IHC), serves as a guidepost for recommending treatment [16]. However, PD-L1 is an imperfect biomarker since protein expression i) is a continuous variable, ii) carries temporal and spatial heterogeneity within the tumor and iii) scoring criteria are not standardized [17]. A significant unmet need in oncology is a robust, simple test to accurately predict patient responses to ICI therapy and their risk of experiencing irAEs.

Other biomarkers currently in clinical use as predictive markers for response to ICIs are tumor mismatch repair deficiency (dMMR), microsatellite instability-high (MSI-H) status and high tumor mutational burden (TMB) [16, 18–20]. The higher mutational loads associated with all three of these states are thought to increase the number of neoantigens expressed by the tumor, thereby increasing potential anti-tumor activity. This set of response biomarkers requires tumor biopsies for DNA extraction, and status is assessed by comprehensive genomic profiling by NGS or targeted whole-exome sequencing. Results have been helpful in decision making even though predictive values are modest [21–25]. Some promising tests under development use RNA extracted from tumor tissue for expression profiling [26, 27] or sequencing of targeted gene panels [28, 29]. Non-sequencing approaches to TME assessments have included measuring T cell diversity [30] or quantifying tumor-infiltrating T lymphocytes [31]. However, tumor tissue is not always available or existent and intratumor heterogeneity is confounding [19]. A liquid biopsy test is now commercially available based on extracting circulating free tumor (cf)DNA from a blood sample and sequencing a panel of genes combined with determining MSI-H and TMB status [32]. This and several other recently developed blood tests show strong concordance with the biopsied tissue-based versions of the same sequencing analyses [32, 33] though modest correlations with ICI response. Other cfDNA sequencing-based approaches being explored rely on microRNA and methylated DNA [34, 35]. In sum, clinical biomarkers correlated with ICI response in either blood or tissue play a role in guiding treatment decisions, but each has limitations [19, 36]. A simple test for accurate prediction of a patient’s benefit from ICI treatment is desirable. Furthermore, a simple test for predicting irAEs is needed, since they increase the risk of therapy related mortality and morbidity [11, 37, 38]. Identifying these patients can help clinicians tailor treatment plans and minimize interruptions, help recognize toxicities early, and prepare mitigation strategies.

We have studied a distinct source of cancer-specific biomarkers: antibodies to neoantigens generated by translation of RNA-level errors. In all cells, errors in RNA transcription and processing occur more than 100-fold more frequently than errors in DNA replication [39]. Error rates in tumors are even further escalated relative to healthy cells, and the normal repair systems are compromised or overwhelmed. Consequently, many of these RNA errors are translated into frameshifted peptide variants. For example, exon-skipping during splicing can create transcripts that result in alternative protein coding frames. Indels resulting from RNA polymerase slippage through microsatellite loci can also create transcripts that will lead to the translation of alternative protein coding frames. Downstream of these variant transcripts, the incorrect amino acids create aberrant C-terminal protein tails. As these are neoantigens, not self-antigens, they are highly likely to elicit T and B cell responses. Frameshift neoantigens (FSN) arising from RNA mis-processing in tumor cells have been shown to generate highly immunogenic peptides in mice and dogs [40].

We have shown that peripheral-blood antibodies to portions of these FSNs, termed frameshift peptides (FSPs), are measurable in cancer patients as well as in mouse tumor models [40–42]. When tumor-bearing mice are vaccinated with FSPs that their tumors express, therapeutically protective responses are achieved [40, 41]. We hypothesized that the diversity of antibodies against FSPs in cancer patients would be sufficiently informative as predictive biomarkers to classify them by future ICI outcomes.

To search this immense biomarker cache, comprising the millions of FSPs a tumor might produce, we have developed a technology to functionally screen 100’s of thousands of them simultaneously. We designed and produced high-density, in situ-synthesis peptide microarrays that display informatically predicted tumor FSPs. Each array contains 374,082 15-mer peptides corresponding to 190,865 predicted frameshift neoantigens that could be generated from (i) exon splicing errors, (ii) exon 1 translational mis-initiation or (iii) transcriptional slippage within microsatellite regions [43]. An out-of-frame translation of an mRNA produces a deviant peptide string. We used these microarrays in massively parallel, competitive serological assays to detect antibodies in patients’ blood that recognize the variant ligands. These quantitative measurements of functional antibody activity are performed on samples collected prior to PD-(L)1 ICI therapy regimens. Here we report on the feasibility of this FSP microarray assay for predicting lung cancer patients’ responses and likelihood of irAE toxicities, in a single test.

## Methods

### Blood collection and processing

Peripheral blood samples were commercially collected and biobanked by Indivumed, Inc. (Hamburg, Germany). All blood samples were collected at baseline, prior to ICI therapy. Tumor radiologic response was determined using the Best Response Evaluation Criteria in Solid Tumors 1.1 (RECIST v1.1). “Responders” are defined as radiologic complete response (CR) and partial response (PR) [44]. “Non-progressors” are defined as responses of CR, PR and also stable disease (SD). Patient irAEs were evaluated as grades (G) 0–4 [45]. All participants provided written informed consent for the collection, and transfer to our labs was approved under IRB Ci-002 from WCG WIRB. Purchased aliquots were received on dry ice and stored at – 80 °C prior to use.

### FSP array serological assay

High density FSP arrays were *in-situ* synthesized on silicon wafers using t-BOC chemistry and photolithography as previously described [43, 46]. The wafers were diced into 13 slides of 75 mm x 25 mm. Each slide contains 16 peptide arrays of 7.67 mm × 7.67 mm, displaying 374,084 peptides. To prepare for the sero-assay, serum samples were diluted 1:50 into an incubation buffer (0.75% casein in phosphate buffered saline with 0.25% tween20, PBST). The diluted sera samples (200µl) were incubated in individual arrays using a gasketed cassette at room temperature for 24 h. Following 3 washes with 1 × PBST, peptide bound antibodies were detected by incubation with 4 nM of Dylight 550 labeled goat anti-human Fc IgG secondary antibody (ThermoFisher Scientific, Cat# SA5-10,135) in 0.75% casein/PBST at 37 °C for 1 h. Slides were washed 3 times with 1 × PBST, twice with dH_2_O and once each with 40% and 100% isopropanol. Slides were dried by centrifuging at 800 RPM for 2 min. The fluorescent signal of bound secondary antibody was detected in an InnoScan 910 laser scanner (Innopsys, France) and the raw relative fluorescent units (RFU) were extracted and tabulated using MAPIX (Innopsys, France) gridding software.

As control for our peptide synthesis platform, the arrays include peptides corresponding to well established monoclonal antibody epitopes and immunogenic peptides from several viruses (HCV, HSV, and HIV). Test arrays are assayed with titrations of i) the corresponding monoclonals or ii) sera-converted samples using the same protocol described above. Strong antibody-dose dependent signal on the cognate epitopes, and only background signal on the non-cognate peptides and FSPs, are confirmed prior to wafer use for sera analyses [46].

### Statistical methods

A total of 74 serum samples from unique patients were analyzed. For the response analyses, patients were required to have participated in immune checkpoint inhibitor therapy for at least 6 weeks prior to radiologic assessments. This excluded 8 samples, leaving 66 for evaluation. Samples were analyzed for response correlations as a full cohort as well as subgroups intended to reduce cohort heterogeneity stemming from patients with stable disease, chemotherapy co-treatment or SCLC subtype diagnosis. For the adverse event analysis, 14 patients of the 74 were removed because irAE annotations were not available. For the remaining 60 samples, all irAEs and the time of event since treatment were recorded.

Serum samples were run in quadruplicate on the FSP arrays as described. For each of the samples, peptides were scored as patient-positive for the presence of antibody binding if at least 2 of the 4 replicate arrays showed signal over 20,000 RFUs or approximately 50-fold above background signal. This returned 5445 peptides from the 374,084 screened. Only these 5445 FSPs were included in the study’s statistical analyses. Across the 66 samples used for the response analysis, 3722 of the 5445 FSPs were patient-positive in 3 or more of the 66 serum samples. Across the 60 samples used for the adverse event analysis, 4209 of the 5445 FSPs were patient-positive in 3 or more of the serum samples. These 3722 and 4209 were the only peptides sufficiently represented in the cohort to be included in the respective chi-square contrast analyses.

The FSP sero-assays do not generate log normal distributions. Although the array readout of fluorescence is continuous, the biological measurements of FSPs are discontinuous. Another characteristic is a sparseness of positive binding readouts. For any single serum sample, only ~ 10^3^ of the peptides on the array will typically display reproducibly positive signals 50-fold above background; the remaining FSPs on the array will be scored negative. This drives distributions that are bimodal and positively skewed. As a result, linear models commonly used to analyze microarray data were not considered optimal.

For the initial step in the analysis, a resampling method was used wherein 80% of the samples were selected without replacement and a chi-square test was performed 100 times. Peptides with *p*-values less than 0.05 in at least 70 of the 100 subsets were retained for the model. Each peptide was assigned as associated with one or the other of a contrast group based on which group had scored more positive samples for the peptide. For example, if a retained peptide had 10 positive samples from responder patients and zero from non-responder patients, the peptide would be assigned as a responder-associated peptide. This example indicates an antibody response preferentially present in responder patients. The peptides were contrast coded as either + 1 or − 1 depending on whether it was associated with responders or non-responders, respectively. If a sample was not scored positive for a peptide, its score for that peptide was zero. The sum of the scores for all model peptides was calculated for each patient sample. This score was used to classify a sample into a predicted outcome group. The summed score for a sample could be zero either because it did not score positive for any of the model peptides or because it was positive for an equal number of peptides associated with both groups of a contrast. These samples were considered indeterminate.

For adverse event modeling, a similar analysis was performed. Cohort resampling of 80% for feature selection were performed at least 100 times. Unlike the response analysis, a unidirectional scoring was used for the holdout samples because the 11 peptides recurrently identified as differentially bound in 70 of the 100 iterations were exclusively associated with the high irAE patient samples. Samples scoring as positively bound for 1 or more of the 11 high irAE-associated peptides were model-predicted to experience an irAE. The response and adverse event analyses were independently repeated for validation.

## Results

A cohort of 74 serum samples from patients with advanced lung cancer were tested, comprised of 86% NSCLC and 14% small cell lung cancer (SCLC). Following blood sample collection, each patient was treated palliatively with either anti-PD-1 or anti-PD-L1 monoclonal antibody as ICI monotherapy (60%) or in combination regimens with chemotherapy (40%). Best radiologic responses were categorized per RECIST v1.1 as CR, PR, SD or PD [44]. Toxicities were categorized as events with guidelines for treatment suspension or re-dosing (G 2–4) or not (G 0, 1) [47, 48]. The distribution of treatment outcome categories across the cohort is shown in Table 1. Individual tumor responses and irAEs during the patients’ time in the trial are reported in Additional file 1: Table S1. Detailed treatment regimens, response assessments, toxicity timepoints, and additional patient clinical information are provided in Additional file 1: Table S2.

**Table 1:** Summary of ICI outcomes within the lung cancer cohort

Best Radiologic Response Category	n (%)
Complete response	1 (1)
Partial response	25 (34)
Stable disease	13 (18)
Progressive disease	27 (36)
Treatment < 6 weeks	8 (11)
Toxicity grade	n (%)
irAE G 0 or 1	42 (57)
irAE G 2–4	18 (24)
Unknown	14 (19)

### Best radiologic response prediction

For developing a model for response to ICI treatment, 8 samples were excluded that had received treatment for less than 6 weeks. The remaining 66 lung cancer patient serum samples were analyzed. These were assigned by best response criteria as non-progressors: complete response, partial response, stable disease (CR/PR/SD, n = 39) and progressors: progressive disease (PD, n = 29). Stable disease patients have been categorized with either responders or non-responders in different studies because assessments are often mixed relative to the response criteria, making these an ambiguous group [49]. For our initial analysis we included SD patients with the responders and therefore this inclusive model more accurately reflects progression versus non-progression of disease.

**Fig. 1:**
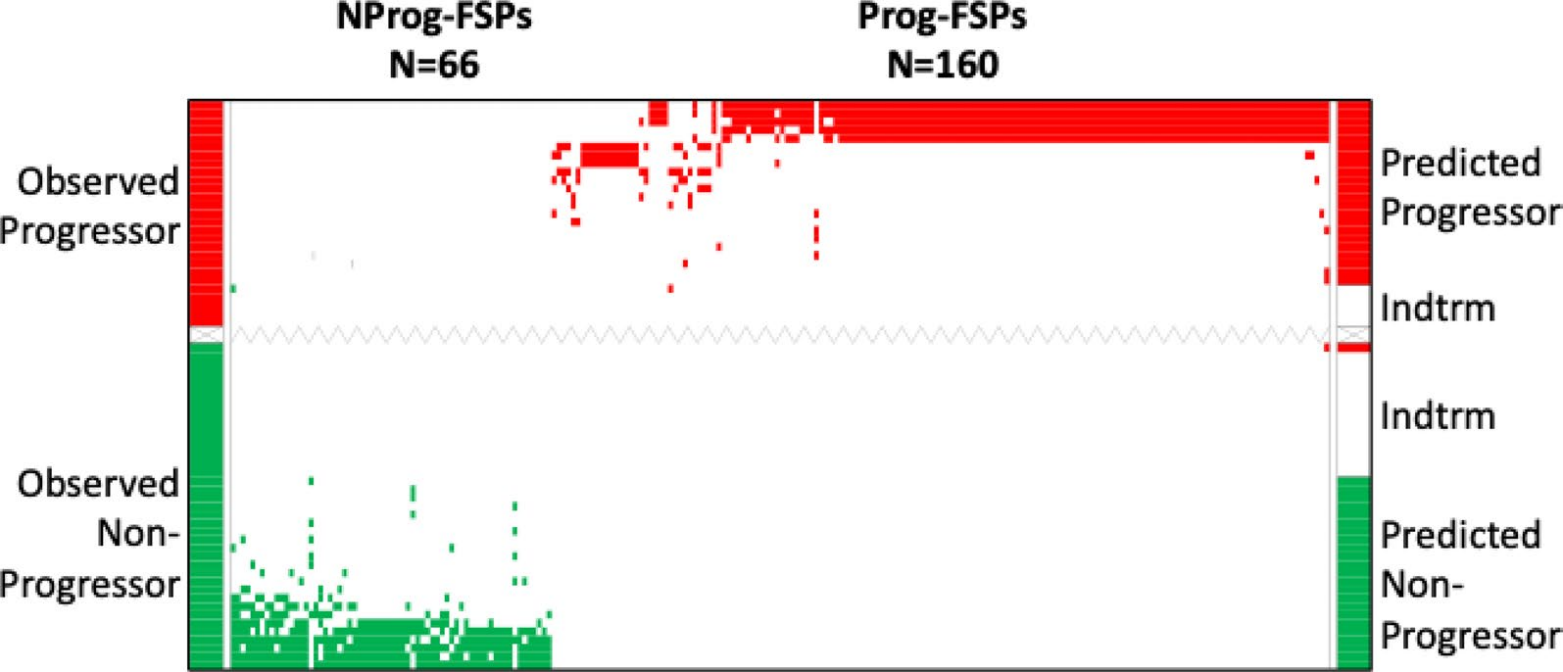
The ordered-scores map displays performance of the predictive model for disease progression compared to observed outcomes. Both observed and predicted non-progressor samples and FSPs are shown in green; observed and predicted progressor samples and FSPs are shown in red; indeterminate samples are in white. FSPs are represented on the X-axis; predicted outcomes of patients are shown on the right Y-axis; observed outcomes of patient are represented on the left Y-axes. *NProg-FSPS* non-progressor associated FSPs; *Prog-FSPs* progressor associated FSPs; *Indtrm* Indeterminate

For model development, 80% of the serum samples were randomly pulled as an analytic sampling set. Chi-square analyses were conducted for differential FSP binding activity between the non-progressors and progressors. Random sampling of another 80% subset and its analysis was repeated 100 times. Using this iterative resampling approach, we identified 66 non-progressor-associated FSPs and 160 progressor-associated FSPs, such that both positive and negative biomarkers of response were being measured (Additional file 1: Table S3). The 226-peptide model was used to calculate an aggregate contrast score for each sample, which would define its predicted outcome group. A sample was classified if it had a non-zero score as described in the Methods. An outcome was predicted for 69.7% of total patients with 97.8% accuracy. The remaining 30.3 of the samples were considered indeterminate because these samples were not bound to any of the model peptides or because their cumulative model binding score was zero. These results are visually depicted in Fig. 1 and tabulated in Table 2. A list of FSPs comprising this model is provided (Additional file 1: Table S5). Two of the serum samples in the cohort were collected from patients with epidermal growth factor receptor (EGFR)-mutant NSCLC, which are clinically observed to be non-responders (or progressors). Consistent with this, the predictive model classified both as progressors. To test the robustness of the resampling approach, the entire analysis was repeated; nearly identical peptides and the similar model metrics were obtained (data not shown).

**Table 2:** Performance matrix of disease progression model

	Predicted Non-progressor	Predicted Progressor	Indeterminate
Observed Non-progressor	23	1	15
Observed Progressor	0	22	5

**Fig. 2:**
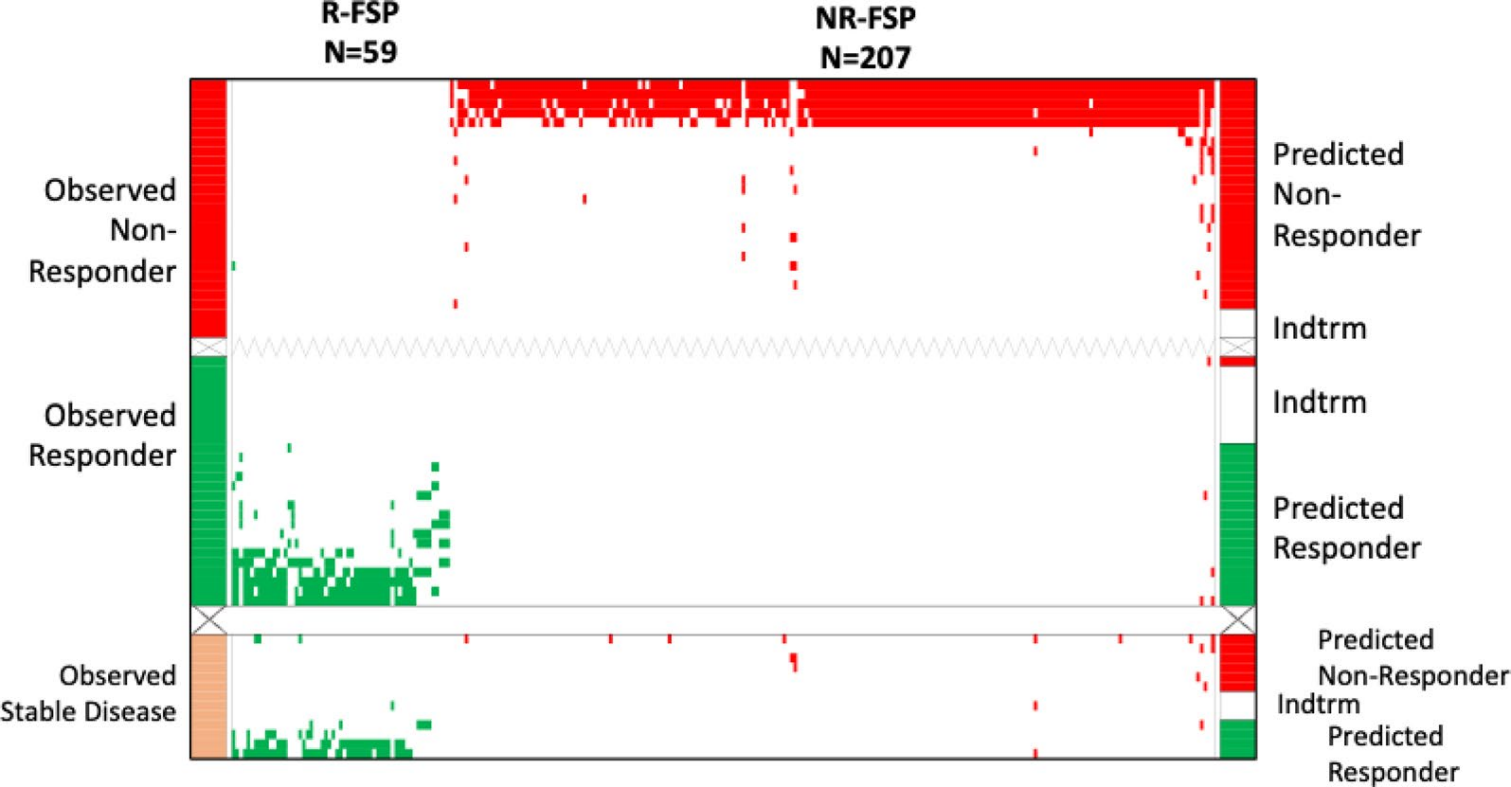
The ordered-scores map displays performance of the model for ICI response trained without SD patient samples. Top panel shows predictions from the peptides derived from the iterated 80% resampling analysis; lower panel displays independent model testing on serum samples from SD patients. Both observed and predicted responder samples and responder associated FSPs are shown in green; observed and predicted non-responder samples and non-responder associated FSPs are shown in red. Observed stable disease samples are shown in tan. Axes labels and other abbreviations are defined in Fig. 1. R-FSP, responder associated FSPs; NR-FSPs, non-responder FSPs

Since SD patients can present with inconsistent readouts for the factors comprising best response criteria [49], we excluded these samples and explored a response model with the remaining 53 samples. We considered this a more clear responder/non-responder contrast. For model development, two-factor chi-square tests were used to identify FSP binding activities associated with responders or non-responders. We found 59 responder-specific FSPs and 207 non-responder-specific FSPs (Additional file 1: Table S4). Of these 266 FSPs, 173 overlapped with the 226 peptides of the all-response model. This less heterogeneous response model was 100% accurate in predicting 78.7% of the samples. This is displayed in Fig. 2, top panel. The same algorithm was next used to predict the 13 pre-treatment samples from patients that would have SD outcomes and had been left out of model building. Four of them were predicted as responders (30%), 6 (47%) as non-responders and 3 (23%) were indeterminate. The distribution of SD patient classification is consistent with the known diversity of their response assessments. This is shown in Fig. 2, lower panel. This response model’s performance is summarized in Table 3 and a list of FSPs comprising the model is provided (Additional file 1: Table S5).

**Table 3:** Performance matrix of response model built on responders and non-responders then tested on stable disease patient samples

	Predicted Responder	Predicted Non-Responder	Indeterminate
Observed Responder	17	1	8
Observed Progressor	0	24	3
*Observed* *Stable Disease*	*4*	*6*	*3*

**Fig. 3:**
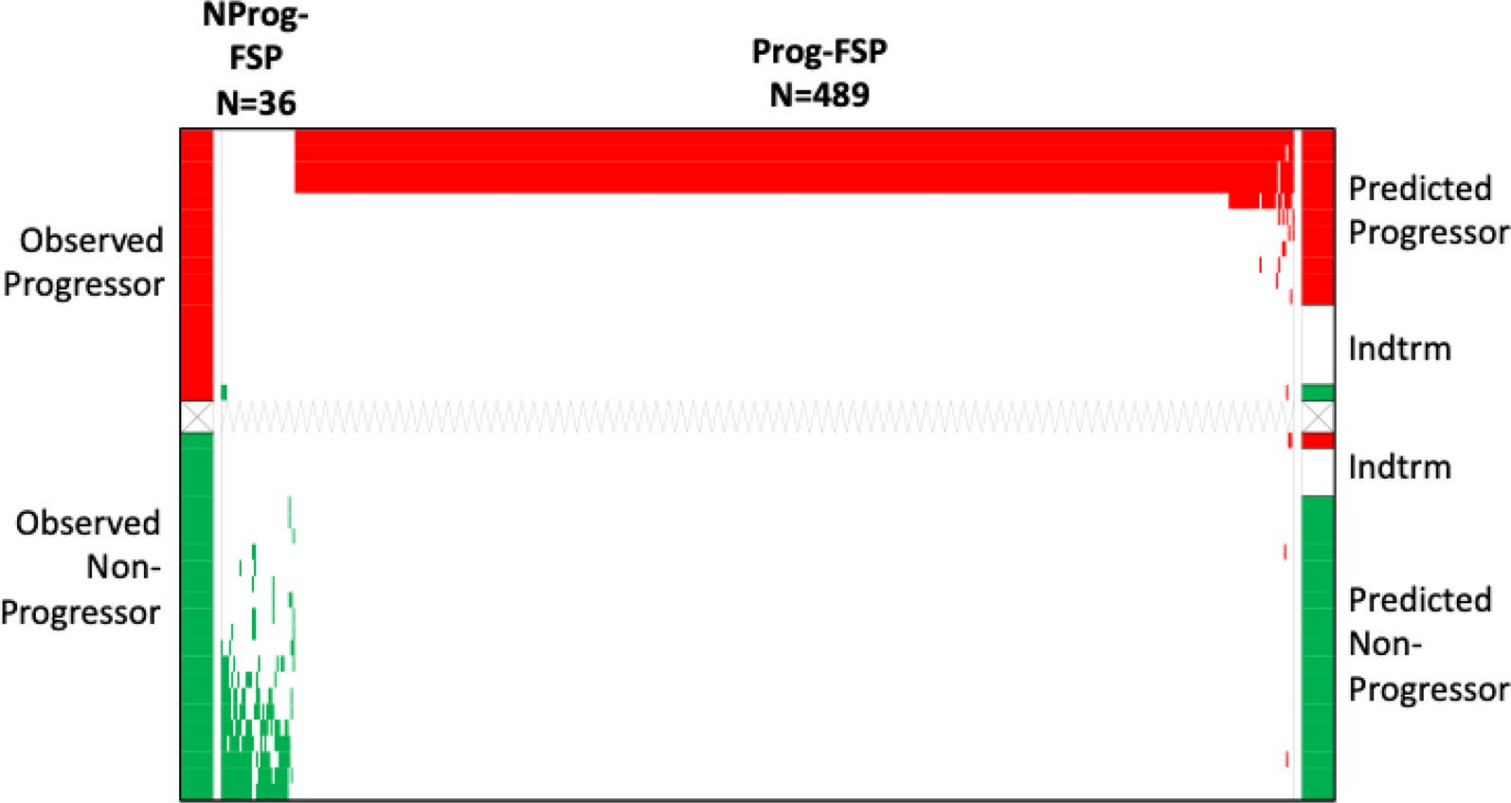
The ordered-scores map displays performance of the model for predicting disease progression following anti-PD-(L)1 monotherapy. Sample color codes, FSP color codes and axes labels and other abbreviations are defined in Fig. 1

Another type of heterogeneity in the 66-patient inclusive responses cohort was treatment regimen. Each received anti-PD-(L)1 but 24 patients also received chemotherapy at the same time (Additional file 1: Table S2). Since there is likely contribution from both components (chemotherapy and immunotherapy) to therapy outcomes, we explored the performance of a model comprised of the 40 monotherapy patients. This model carried a significantly greater number of informative peptides (525 versus 226), with 125 overlapping with the 66 sample inclusive response model. Despite its basis on a smaller cohort, prediction accuracy was 93.8% and 80% of the samples could be classified, as visualized in Fig. 3. The results are detailed in Table 4 and a list of FSPs comprising this model is provided (Additional file 1: Table S5).

**Table 4:** Performance matrix of predictive model of disease progression in patients to be treated with ICI monotherapy regimens

	Predicted non-progressor	Predicted progressor	Indeterminate
Observed non-progressor	19	1	3
Observed Progressor	1	11	5

SCLC is histologically and clinically distinct from NSCLC [50]. Our 66-patient cohort included 9 with SCLC. To explore the impact of subtype heterogeneity, we built a model with the 57 NSCLC patient samples. This model carried a similar number of peptides (281) relative to the full cohort model, was 100% accurate and showed a marginal improvement in sample classification coverage (73.2%). The list of FSPs comprising this model is provided (Additional file 1: Table S6).

**Fig. 4:**
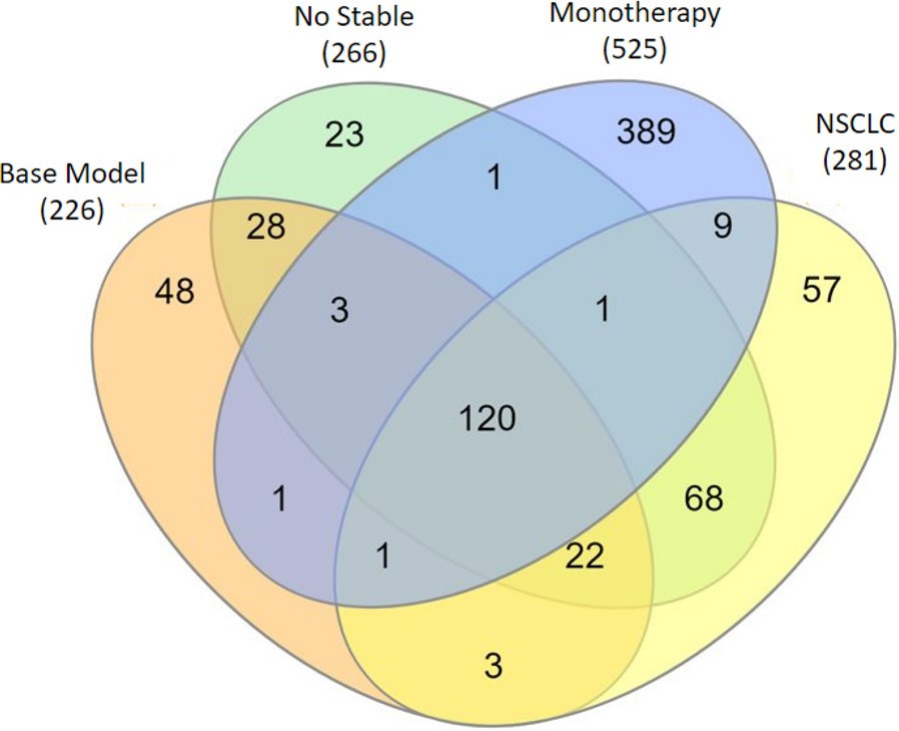
Venn diagram of FSPs shared across 4 models of outcome prediction following ICI therapy. Parenthetic numbers indicate model sizes. numbers within venn petals indicate unique versus overlapping FSPs among the models

The Venn diagram in Fig. 4 displays the number of overlapping and unique FSPs comprising these 4 models of predicted response outcomes. There are 120 peptides shared among all of them; the monotherapy model has the largest number (389) of unique classifying peptides.

In summary these results show that accurate predictive models of ICI treatment response can be built using differential binding profiles of anti-FSP antibodies queried against their peptide ligands. Reducing the heterogeneity of the patient samples with respect to radiologic response criteria, therapy complexity or cancer subtype increased the proportion of samples that can be classified.

### irAE prediction

**Fig. 5:**
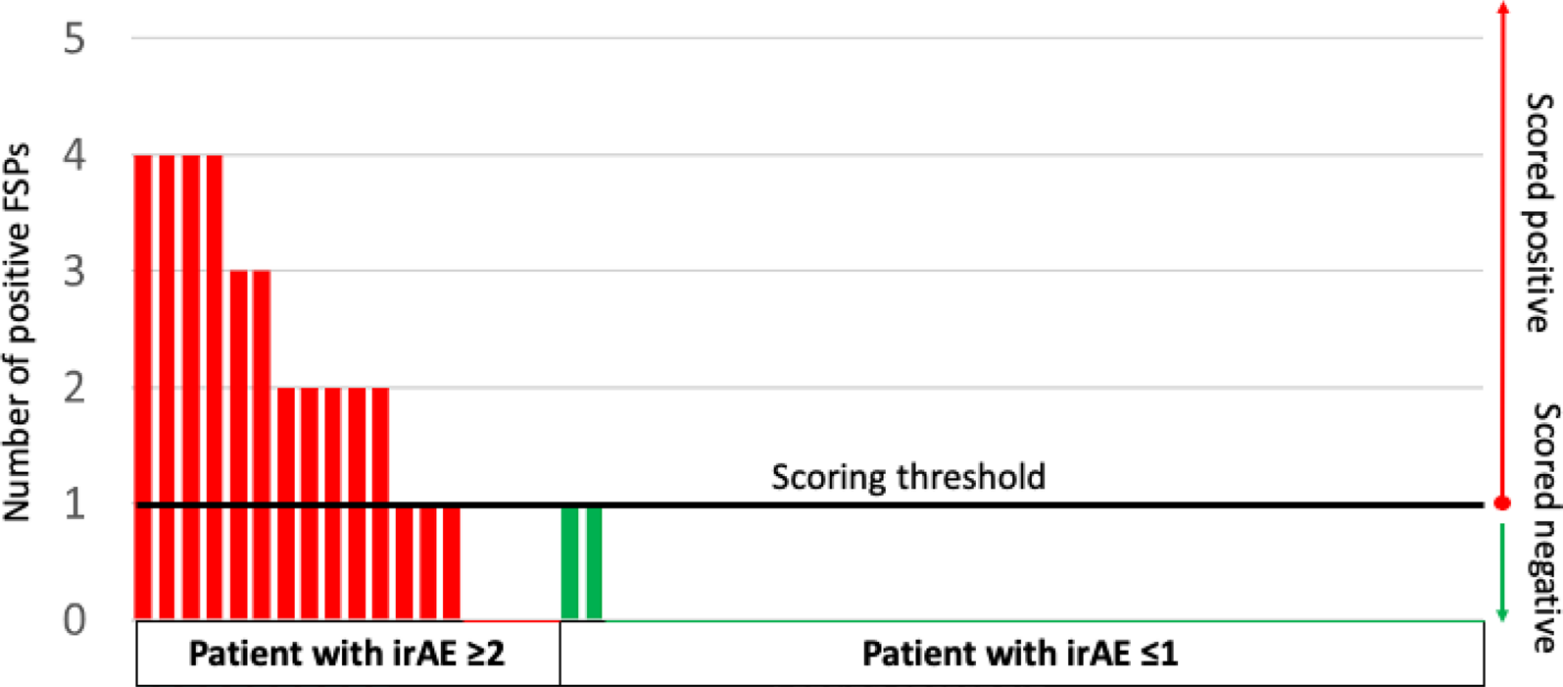
Bar graph displays ordered, positive contrast scores for irAE prediction. A set of 11 irAE-specific FSPs were statistically selected to build a model for irAE prediction. Patients with 1 or more positive FSPs (left Y axis) are predicted to have irAE ≥ G 2. Red bar: patient with observed irAE ≥ G 2. Green bar: patient with observed irAE = G 0 or G 1. Black line: cut-off score for irAE ≥ G 2 prediction. Predictions are shown on right Y-axis

For developing a predictive model for irAE, we grouped clinically symptomatic irAEs (G 2–4) for the development of irAE prediction model. This is the population of patients for whom treatment modifications could be recommended if anticipated. Of the 60 patient samples for which we had irAE annotation, 18 patients were reported to have had irAEs of G 2–4 and 42 patients had G 0 or 1. We performed the same chi-square scoring on 80% resampling without replacement at least 100 times. We identified 11 irAE classifying peptides which were recurrent in at least 70 of the 100 resampling iterations (Additional file 1: Table S7), and all of these were exclusively positive in the symptomatic irAE group. This diverges from the response to treatment analyses in which there was a balance of positive FSPs correlated with both groups of a contrast (e.g. responders and non-responders). This unidirectional structure of the FSP binding activity suggested a one-sided scoring approach could be used rather than the two-sided scoring with indeterminates. The 60 samples were contrast-scored against the 11 irAE-specific FSPs. Patient samples with a threshold number of positive model FSPs were classified into the symptomatic irAE group with 87.5% sensitivity and 91% specificity. Samples from 7 patients with observed symptomatic (G 2–4) events did not meet the threshold for positive model peptides. Since these were defaulted to the asymptomatic (G 0,1) irAE group, prediction accuracy was 90% with no indeterminates. To confirm robustness, the entire resampling analysis was repeated, and similar results were achieved. In Fig. 5, these results are displayed in a bar graph. This representation highlights the one-sidedness of positive features and allows the samples to be ordered by their prediction score. Table 5 shows the 2 × 2 prediction model matrix. None of the irAE model peptides overlapped with any of those in the tumor response models.

**Table 5:** Performance matrix of predictive model for irAEs

	Predicted symptomatic irAE	No predicted symptomatic irAE
Observed symptomatic irAE	14	4
No observed symptomatic irAE​	2	40

For the irAEs, we were unable to explore the impact of more homogeneous lung cancer subtypes or treatments because holding out the corresponding samples reduced the symptomatic irAE group size below that required for a statistically meaningful analysis.

In summary these results show that the same antibody binding profiles established on the FSP arrays used for best response prediction can also be analyzed for developing a predictive model for experiencing irAEs.

### Informatic analysis

For the full cohort, all-response model, a mapping analysis of the 226 informative peptides to the human reference genome was conducted. We found the informatically predicted RNA-error derived neoantigens of 3 genes were each the source of 2 different classifying-FSPs. For 1 of these 3 neoantigen source genes, its 2 associated classifying-FSPs mapped to different parts of the same predicted neoantigen (Table 6). The positive scoring of multiple FSPs derived from RNA-errors in the same genes is consistent with a biological relevance to the antibody measurements.

**Table 6:** Response-classifying FSPs from neoantigens predicted from the same genes

Response FSP	FSP related gene
PAPGEPWEAGGPHAG	DOK7^a^
PPPAFFSACPVCGGL	
PAPFWPARPLLSAGI	CBARP
PAPLQALLGRPPAPQ	
GPPPEEAADGTAASN	FDXR
PPGPRGHLRETACAL	

^a^2 classifying FSPs map to the same predicted frameshift neoantigen of this gene

A gene ontology (GO) Oncology analysis was used to assess whether there was any enriched molecular or cellular function or components in the 224 source genes corresponding to the mRNA mis-processing events that would have produced the 226 classifying FSPs in the full-cohort response model. No enriched pathway was found. However, the source genes of some of the FSPs used to build the predictive models of clear response-only (no SD) and monotherapy-only (no combination therapy) were enriched in a few pathways (Additional file 1: Table S8).

The FSPs are predicted as tumor cell errors, however the frameshifted variants generate random peptides that might share identity or similarity with a self-protein and potentially induce an autoimmune response. The 11 FSPs predictive of G 2–4 irAEs were blasted to the latest (08/19/2022) reference protein sequences of the GRCh38 with local BLAST + under the default setting of blastp-short. Four of these classifying FSPs carry sequence strings of 8–10 amino acids with at least 80% identity to 7 self-proteins, including 1 FSP (DPPASASQ) with 100% identity to 4 proteins (Table 7). A GO Oncology analysis of these 7 genes showed no biological enrichments. However, most of these genes are preferentially expressed in organs often affected by ICI toxicities such as lung, colon and testis [51] (Additional file 1: Table S9). Additional serum samples would be needed to investigate any correlations between organ-specific gene expression and toxicities.

**Table 7:** Toxicity-classifying FSPs with self-protein homology

Protein fragment	Identity (%)	Fragment length	Protein name	Protein ID
DPPASASQ	100	8	DNA (cytosine-5)-methyltransferase 1 (DNMT1)	NP_001124295.1
				NP_001305660.1
			nuclear valosin-containing protein-like (NVL)	NP_001230075.1
				XP_016856867.1
				XP_047277577.1
				XP_047277591.1
				XP_016856869.1
				XP_011542498.1
				XP_016856873.1
				XP_047277569.1
				XP_016856874.1
			rab5 GDP/GTP exchange factor (RABGEF1)	NP_001354672.1
				NP_001354673.1
			SEC14-like protein 4 (SEC14L4)	XP_047297303.1
				XP_047297304.1
LSPSARPRS	88.9	9	septin-4 (SEPTIN4)	NP_001185642.1
				NP_001243711.1
				NP_001355701.1
				NP_004565.1
				NP_536340.1
				NP_536341.1
				XP_006722012.1
				XP_047292265.1
ESRARRSSYA	80	10	phospholipid-transporting ATPase IK (ATP8B3)	NP_001171473.1
				NP_620168.1
NLLRPEVR	87.5	8	ECT2L epithelial cell transforming 2 like (ECT2L)	NP_001071174.1
				NP_001181966.1
				XP_006715535.1
				XP_011534097.1
				XP_011534099.1
				XP_016866317.1
				XP_016866318.1
				XP_016866319.1

These informatic analyses show some FSP sequences that are ligands for response outcome-associated antibodies correspond to variant transcripts of the same genes. FSPs that are ligands for irAE-associated antibodies share similarity with several self-proteins. Altogether, this study indicates that antibodies to frameshift neoantigens can serve as predictors of checkpoint inhibitor treatment outcomes relative to disease progression, tumor response, therapy complexity and adverse events.

## Discussion

**Fig. 6:**
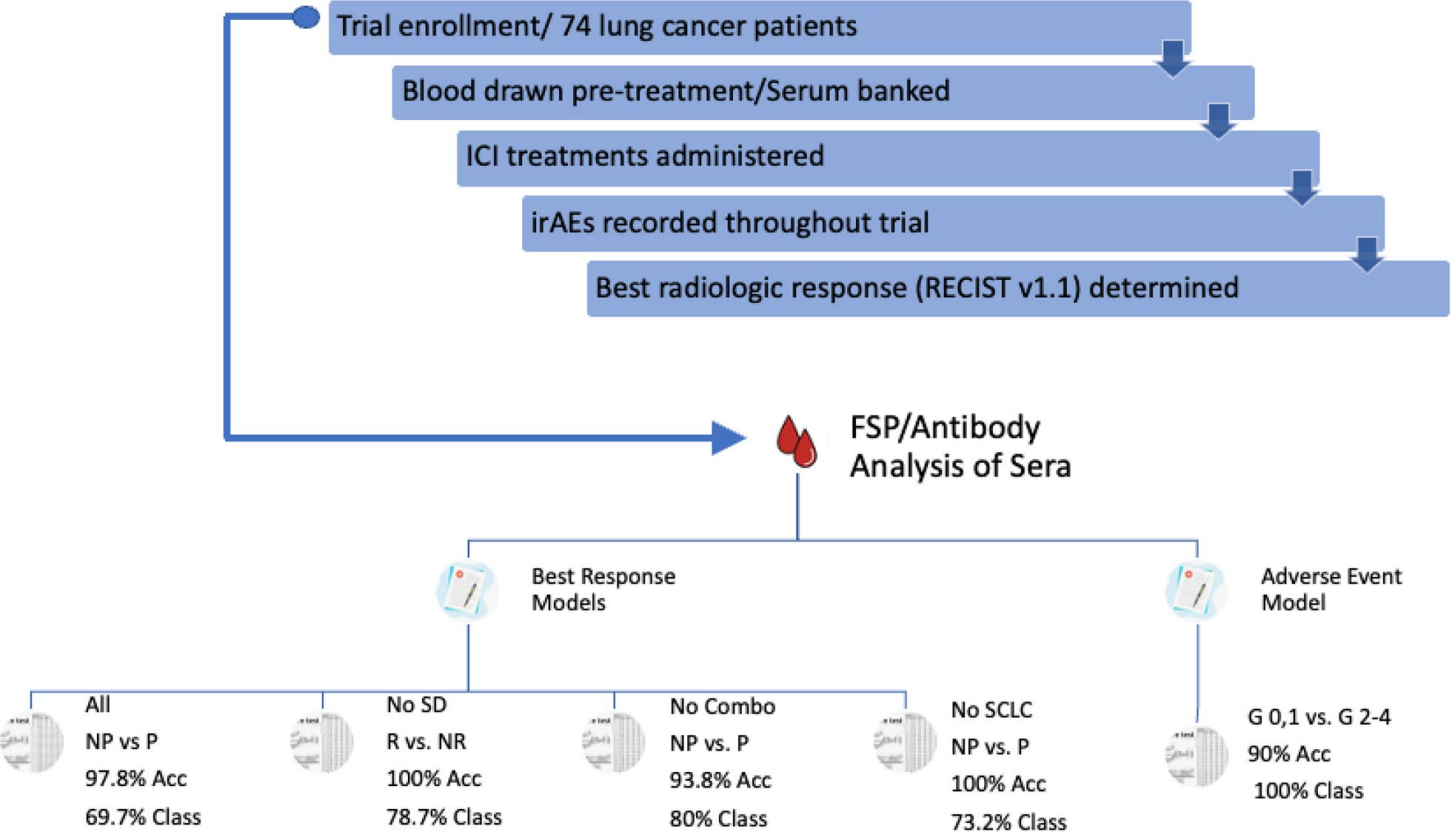
A checkpoint inhibitor trial enrolled 74 patients diagnosed with advanced lung cancer. Venous blood draws were collected followed by initiation of their ICI treatment regimen. Adverse events were continuously observed; tumor responses were regularly monitored. Best radiologic response was used as the observed response for analysis in our study. The serum samples collected pretreatment were analyzed on the FSP/Antibody assays to develop models of predicted response and adverse event. *NP* non-progressor, *P* progressor, *R* responder, *NR* non-responder, *Acc* accuracy, *Class* classified

In overview, our feasibility study demonstrates the utility of a new class of blood biomarkers, anti-FSP antibodies, that can be measured by probing sera against large libraries of novel frameshift neoantigens synthesized as peptides on a silicon wafer. The presented study uses blood samples collected from a cohort of 74 lung cancer patients before receiving PD-(L)1 immunotherapy. Antibody biomarkers were used to build models to predict therapy outcomes. All 4 response models that were built predicted outcomes with nearly complete accuracy. However, the proportion of a cohort that could be predicted by a model was influenced by the patient heterogeneity. Using the same binding assay data, a model was developed to predict adverse immune events that displayed 90% accuracy with all samples predicted. Some of these predictive FSPs show similarity to self-proteins. A study flow chart and summary of the models with their performances are presented in Fig. 6.

For developing the first, most heterogeneous predictive model of ICI response, sera from the 66 patients who were treated for at least 6 weeks were analyzed. Differential anti-FSP antibody activities were identified on the FSP arrays that correlated with future outcomes. We demonstrated that 226 FSPs preferentially bound by an outcome group could predict response with 97.8% accuracy in the patient samples showing cumulative non-zero model scores (69.7%). Within the study cohort there were 2 EGFR-mutant NSCLC patients. Compared with EGFR wild-type tumors, those with EGFR mutations show more heterogeneity in the expression level of PD-L1, TMB, and other TME characteristics. Trials have shown no response to ICI treatments in NSCLC patients with EGFR-mutant tumors [52]. Here we show that the EGFR-mutant patients were classified as future progressors, consistent with their historically observed non-responsive outcomes.

A second response model was built in which the SD patient samples were excluded. We anticipated that building a model with only samples from patients with clear response outcomes (not stable disease) might increase the number of positively-bound FSPs shared within a group and the number differentially bound between the groups. Accuracy and model size were similar and as hypothesized, the proportion of samples classified, with non-zero model scores, increased to 78.7%. This model was applied to the SD samples excluded from model building; their classifications showed a distribution between the observed outcome groups. These results are consistent with their ambiguous response characteristics.

In a third model, patients who had received ICI in combination with chemotherapy were excluded. Accuracy was only marginally reduced, even though this was the smallest sample cohort size of the models tested. Outcome-associated model-peptides increased more than twofold and the proportion of predicted samples increased to 80%. The larger number of outcome-associated FSPs likely contributed to the reduction in indeterminates. We might hypothesize that the pre-existing immune profiles unleashed by anti-PD-(L)1 are different than those unleashed by anti-PD-(L)1 plus secondary therapies. Therefore, the number of shared biomarkers within any outcome group would be less frequent in the combination cohort versus the monotherapy one. Accurate prediction of monotherapy response might guide a decision for not pursuing a chemotherapy combination regimen.

In a final model, samples from patients with the more aggressive SCLC subtype were excluded. This model of response prediction, built with only NSCLC patient samples, showed high 100% accuracy in predicted 73.2% of the samples. This was the smallest increase in sample coverage relative to the original full cohort model, suggesting that the immune heterogeneity of these clinically very different lung cancers [50] are not greatly different.

We anticipated that the response models would consist of anti-FSP binding activity present in samples of patients with positive response outcomes and absent from the other. This would be consistent with generating anti-tumor immune responses that had been unsuppressed by ICIs. However, in all 4 models we observed differential binding events associated with both the positive and negative groups of the response contrasts. The negative group- associated antibodies might be correlated with ineffective immune activities. Alternatively, these FSPs correspond to inhibigens, which have been noted as neoantigens that actively have suppressive effects on T -cell responses [53].

The GO Oncology enrichment analyses of the models in this study did not reveal any strong pathway themes. This suggests that the differences in antibody binding activity in both the response and toxicity contrasts are not predominantly driven by gene transcript mistakes in functionally related proteins or pathways. Instead, it may be a characteristic of how the source genes’ transcription or transcript processing is recurrently altered in tumor cells. This hypothesis fits well with the observation that variant versions of 3 gene transcripts were each the source of more than one FSP in the full cohort, all-response model.

A variety of molecular biomarkers have been studied toward developing a test for predicting patient responses to ICI therapeutics. Some recent approaches have shown encouraging predictive value; however, biomarker extraction or testing is elaborate, and sometimes not possible. By contrast, antibodies are immune effectors and therefore they directly read out immune activity. Antibody biomarkers are massively amplified by B cells that have been activated because of their recognition of tumors. This specific amplification of the desired biomarker facilitates their measurement. In general support of the utility of antibodies as biomarkers, a very recent study showed that antibodies against self-antigens can serve as biomarkers of melanoma recurrence following adjuvant therapy [54]. The antibody biomarkers against non-self, neoantigens described here are additionally unique measurements of patient status. For example, they are elicited by a diverse and plentiful source of frameshifted peptide variants that exist in tumors from the translation of RNA-level mistakes. These out-of-frame variants occur at high frequencies in tumor cells because of the relaxation of normal processing and editing systems. Frameshift peptides are more likely to be immunogenic since the full peptide sequence is variant versus a single amino acid change within an otherwise normal sequence, or versus self. Furthermore, our sero-assay measures IgG antibodies, which require CD4 T-cell help for affinity maturation. Therefore, these T cells are implicated as direct players in anti-tumor immune responses. In sum, these abundant, immunogenic biomarkers are useful harbingers of future patient outcomes.

We also investigated whether the anti-FSP antibody biomarkers could predict which patients would experience irAEs. An earlier study supports this possibility. The autoimmune antibody marker rheumatoid factor was found significantly correlated with patients who would subsequently experience irAEs [55]. We considered that anti-FSP immune activity might cause autoimmunity in the host even though FSPs are variants and therefore presumably non-self. However, some of these frameshift variants might have homology to normal host proteins and serve as unintended mimotopes of self, even though we had removed peptides from the library design with high homology. By contrast, the FSPs comprising the response models are preferentially associated with immune responses to the “foreign” tumor and therefore likely to be distinct from self. This hypothesis is consistent with observed lack of overlap in classifying FSPs between the response and toxicity models.

Our model provided 90% accuracy in predicting toxicity with no indeterminates. All 11 FSPs selected for building this predictive model were associated with the symptomatic irAE group. This bias in group-associated peptides is not what was observed in the response models but is consistent with ICIs stimulating an immune response against self in the toxicity group, which would be absent in the non-toxicity group. A recent study showed that T-cells recognizing an epitope of Napsin A are associated with irAEs in lung cancer [56]. In our study, we found that 5 of the 11 predictive FSPs showed significant homology to stretches of host proteins.

Identifying patients that may experience untoward autoimmune events is not likely to rule them out for ICI therapy because irAEs have been linked to some positive tumor responses [19, 57]. Instead, these patients would be closely monitored during the treatment schedule, with possible suspension or alternative dosing. Recent studies indicate the correlation between irAEs and response may be complex [37]. For instance, the presumed cross-reactivity between tumor and host tissue appears to be limited to late onset events (> 3 months following treatment) or those related to certain tissues [57, 58]. These results suggest a distinct biology between irAEs of different onset timing or development sites. We did not have sufficient sample numbers from patients with irAEs to analytically stratify these clinical subgroups. Another limitation of this study is that we had filtered out peptides from the library design with strong homology to the proteome. Consequently, the FSPs with the highest likelihood of autoimmune activity would not have been assayed here. Though accurate, this may explain the small number of FSPs comprising the irAE predictive model.

The small, retrospective collection of samples from a clinically heterogeneous cohort of lung cancer patients limits this study. It is also limited by the incompleteness of the FSP library used here. Less than 20% of the possible 2.1 million FSP antibody-ligands were included in these arrays. Test accuracy was high, but a proportion of the samples were not classified because, among the peptides measured on the array, these sera did not show differentially bound FSPs that were shared by those of the rest of the cohort. Expanded, prospective sample collections queried on more comprehensive FSP arrays will enable validation of these results and increase the number of informative FSPs for each subgroup. This is anticipated to provide for significantly higher cohort coverage and more detailed evaluations of clinical and treatment subgroups. This test might also be combined with another liquid biopsy test to ensure full patient coverage.

## Conclusions

In summary, we present the feasibility of a simple, accurate, serological test that uses a small amount of blood to predict ICI outcomes. It works by measuring a new class of biomarkers (anti-FSN antibodies) that are sufficiently comprehensive to inform both future ICI clinical responses and toxicities. These biomarkers represent an orthogonal determination of patient status relative to others explored for predicting response, and a new one for predicting irAEs. Future efforts will focus on improving this approach for addressing specific needs in lung cancer treatment and for evaluating this ICI predictive test platform for other cancers.

## Supplementary Information

**Additional file 1: Table S1.** ICI outcomes assigned to each study sample. **Table S2.** Detailed lung cancer cohort patient description. **Table S3.** Classifying FSPs in full cohort all-response model. **Table S4.** Classifying FSPs in response model without stable disease. **Table S5.** Classifying FSPs in monotherapy response model. **Table S6.** Classifying FSPs in NSCLC response model. **Table S7.** Classifying FSPs in adverse event model. **Table S8.** GO analysis of source genes for the classifying FSPs comprising the response without SD and monotherapy response models. **Table S9.** Expression levels* of source genes for irAE-classifying FSPs.

## Data Availability

Data generated or analyzed during this study are included in this published article, its supplementary information files or are available from the corresponding author upon reasonable request.

## Abbreviations

ICI: Immune checkpoint inhibitor
PD-1: Programmed cell death receptor
PD-L1: Programmed cell death receptor-ligand 1
CTLA-4: Cytotoxic T-lymphocyte-associated protein-4
irAE: Immune-related adverse event
FSP: Frameshift peptide
FSN: Frameshift neoantigen
NSCLC: Non-small cell lung cancer
mNSCLC: Metastatic NSCLC
SCLC: Small cell lung cancer
IHC: Immunohistochemistry
dMMR: Mismatch repair deficiency
MSI-H: Microsatellite instability-high
TMB: Tumor mutational burden
TME: Tumor microenvironment
VEGF: Vascular endothelial growth factor
RECIST v1.1: Best response evaluation criteria in solid tumors 1.1
CR: Complete response
PR: Partial response
SD: Stable disease
PD: Progressive disease
cfDNA: Cell-free DNA
EGFR: Epidermal growth factor receptor
GO: Gene ontology
