# Supplementary material for: Predicting response and toxicity to immune checkpoint inhibitors in lung cancer using antibodies to frameshift neoantigens
Source: J Transl Med. 2023 May 22;21:338. doi: 10.1186/s12967-023-04172-w (PMC10201711; doi:10.1186/s12967-023-04172-w)
Supplement: Supplementary file 1 — Additional file 1: Table S1. ICI outcomes assigned to each study sample. Table S2. Detailed lung cancer cohort patient description. Table S3. Classifying FSPs in full cohort all-response model. Table S4. Classifying FSPs in response model without stable disease. Table S5. Classifying FSPs in monotherapy response model. Table S6. Classifying FSPs in NSCLC response model. Table S7. Classifying FSPs in adverse event model. Table S8. GO analysis of source genes for the classifying FSPs comprising the response without SD and monotherapy response models. Table S9. Expression levels* of source genes for irAE-classifying FSPs. [file 12967_2023_4172_MOESM1_ESM.zip › Additional file/Supplementary Tables S1,3 to 9.docx]

**Additional file 1: Table S1. ICI outcomes assigned to each study sample**

| **Patient ID** | **Best Response*** | **irAE Grade**** |
| --- | --- | --- |
| CAL_001 | PR | N/A |
| CAL_003 | PD | 0 |
| CAL_004 | PD | 0 |
| CAL_005 | SD | N/A |
| CAL_006 | PD | N/A |
| CAL_007 | PR | N/A |
| CAL_008 | PD | 0 |
| CAL_009 | SD | 0 |
| CAL_010 | PR | 0 |
| CAL_011 | PD | 0 |
| CAL_012 | PD | N/A |
| CAL_013 | PD | N/A |
| CAL_014 | SD | N/A |
| CAL_015 | SD | N/A |
| CAL_016 | PR | N/A |
| CAL_018 | PR | 0 |
| CAL_019 | PR | 0 |
| CAL_020 | PR | 0 |
| CAL_021 | PD | 0 |
| CAL_022 | PR | 2 |
| CAL_023 | SD | 0 |
| CAL_024 | SD | 0 |
| CAL_026 | SD | 0 |
| CAL_027 | PR | 0 |
| CAL_029 | PD | 0 |
| CAL_031 | PD | 0 |
| CAL_032 | SD | 0 |
| CAL_033 | PR | 0 |
| CAL_034 | SD | 0 |
| CAL_035 | PD | 1 |
| CAL_036 | PR | 0 |
| CAL_037 | PD | 0 |
| CAL_038 | PR | 0 |
| CAL_039 | PD | 0 |
| CAL_040 | PR | 2 |
| CAL_042 | SD | 0 |
| CAL_043 | PD | 2 |
| CAL_044 | PR | 0 |
| CAL_045 | SD | 2 |
| CAL_046 | PD | 0 |
| CAL_047 | N/A | 0 |
| CAL_049 | N/A | 2 |
| CAL_050 | SD | 2 |
| CAL_051 | PD | 2 |
| CAL_052 | PR | 2 |
| CAL_054 | PD | 2 |
| CAL_055 | PD | 0 |
| CAL_056 | PD | 3 |
| CAL_057 | PR | 2 |
| CAL_058 | PR | 2 |
| CAL_059 | PD | 0 |
| CAL_060 | PD | 0 |
| CAL_061 | PD | 0 |
| CAL_062 | PR | 2 |
| CAL_063 | SD | 0 |
| CAL_064 | PR | 4 |
| CAL_066 | PD | 1 |
| CAL_067 | PD | 1 |
| CAL_069 | PD | 2 |
| CAL_070 | CR | N/A |
| CAL_071 | PR | 2 |
| CAL_072 | PD | 2 |
| CAL_073 | PR | 1 |
| CAL_074 | PR | 1 |
| CAL_075 | PR | 0 |
| CAL_077 | PR | 0 |
| CAL_078 | PR | 0 |
| CAL_079 | PD | 0 |
| CAL_025 | N/A | 1 |
| CAL_053 | N/A | 3 |
| CAL_017 | N/A | N/A |
| CAL_002 | N/A | N/A |
| CAL_030 | N/A | N/A |
| CAL_048 | N/A | N/A |

*N/A in Best Response column indicates sample from a patient that did not remain on treatment for at least 6 weeks

**N/A in irAE column indicates sample from a patient with no irAE information provided by the vendor

**Additional file 1: Table S2. Detailed lung cancer cohort patient description**

See excel file

* Column I definitions

PDL1 = negative: No expression, <1%

PDL1 = positive: >1% up to 100% (positive is used very widely for any expression higher than 1%- some clinics do not differentiate between weakly positive and strongly positive)

PDL1 = weakly positive: Low expression 1-49%

PDL1 = strongly positive: High expression >50%

**Additional file 1: Table S3. Classifying FSPs in full cohort all-response model**

| AAAAAGAGGPGGGDA |
| --- |
| ADRGEDGHDPGWPIA |
| ADSSERLSSTQPAHF |
| ADSSGPFRPHVQFIC |
| AHSSRTFKMQILPES |
| AIAWCWPPAAPTSGR |
| AILGKREKKEIQCSF |
| ALGAAPSRAGRGRPG |
| ANVPPGLLLGSLRAH |
| APAPAPDPAAPPARA |
| APAPRLQSARIPGRG |
| APLAGPPCTRLCTRP |
| APLLSASASTSLPSP |
| APLPFTPNRDRCSWQ |
| APLPSSWRPGTGTTI |
| APLPTPRLREVPHRF |
| APPPAGPAAAAAPRP |
| APPPALGTIFSCSPL |
| APPPCRPRVTPGPPP |
| APPPGPQLLWLCGLH |
| APPPPGGPGQAGDPA |
| APPPPPGFRCCAVSG |
| APPPVHTAPAGRGGP |
| APSPPRPRRRPTPTS |
| APVGAEGAAGSRLRP |
| APVPKSLHGGRVCLP |
| APVPPGPAACCCPHR |
| APVPRVCRDTATWFS |
| APVPSHQPVRRAAAQ |
| APVRTTAWASASIID |
| APVVPEASPRRALLP |
| AQLGPKGTMALLENL |
| ARLKRKYHGPLSTMQ |
| ARMSRRTRGPVERAA |
| ARRERRPRVSRLLTP |
| ARRQRRREGGKERGA |
| ARRRRGARGGPGAVP |
| ARRRRGLRTPASVPI |
| ARRRRGQWHAPKETP |
| ARRRRRGPRAPNRPP |
| ARRRRRRHHHQRGDA |
| ARRRRRSPSGSARGP |
| ARRRRRVEHHAAVPT |
| ARRRRSARPSGTARA |
| ARRRRTRSVSRLPQG |
| ASADAAGGEAAHDGH |
| ASADARPPWRSYRRC |
| ATSLLGTREPDTEVI |
| FSSSAPESEISTTIP |
| GAAPTASVSRPGVGG |
| GAAVPQDTRLARLHA |
| GALPREREKSRLCIH |
| GALPRGPAVHQVPAA |
| GALPSADAGLAGGAA |
| GALPSQEGGGAGSRG |
| GAPAHPHRREALRLP |
| GAPALPLGHGDLPWQ |
| GAPAPPCSWTPPQQR |
| GAPAPSGEGRGTSHP |
| GAPISRPCFGCSVES |
| GAPITSSRRRCPCRP |
| GAPLARQARQGLLLD |
| GAPLHMSPGTSHLSA |
| GAPLHPGHPCAGPGH |
| GAPLHPRLHLVAALP |
| GAPLSLSPQRMHPLA |
| GAPLSPTSQSACTLA |
| GAPLVPAPHLHLWPH |
| GAPPEPATAAARSPD |
| GAPTEPSPTGRARGQ |
| GAPVASQRRRRRRRS |
| GAPVATLLRSTLGRN |
| GAPVCRNGTTSCAGR |
| GAPVFPQVYGSGVTC |
| GAPVGGPWRHPYLHA |
| GAPVHPAWHHRDPGG |
| GAPVRRGCSQGPEQR |
| GAPVSGLPEGHAEAG |
| GGRGGGGGRGARLLG |
| GPLPCPHHHWRFAES |
| GPLPGELRSCSTPRR |
| GPLPVLHHQVRGRGF |
| GPPAVAPLVAGQPGL |
| GPPNALAQPRPPGWS |
| GPPPEEAADGTAASN |
| GPPPPAGRGDPARGG |
| GPRGARRPRPPQSAA |
| GPTPLGCRCPAAPFS |
| GPVNILPPSWSSVPL |
| GPVPHGGSPWVGEAL |
| GPVPMRRVSELPGTL |
| NGAPHLMGSGAVSSW |
| NPSAPSATVSPPCAV |
| NPSPGHCQMVQRPEW |
| NPSPHFRDFGHASSS |
| NPSPSCVQPSLPWLS |
| NSPPAAPSVPPAFQG |
| NTAPPTQSLGLAVHP |
| NVANLEKPLDLNKLY |
| PAAAAADHGLGRRFG |
| PAAAAVSGFCHQSLW |
| PAAAPARPWAERPRS |
| PAAAPLHQGRLAASD |
| PAAAPSSACRNSSST |
| PAALPALRVAGCGGL |
| PAALPDAAAGGGARR |
| PAARVPLLPPEPLPR |
| PAARVSPPWLRRSSA |
| PAAWENCLNARGSLP |
| PAAYPPDCPLWLQGC |
| PAAYSSSSCLAAWAR |
| PAGNFHCGARRGESL |
| PAGPAGHAHAAQPGA |
| PAGPSCQILLLSPAT |
| PAGWSPSAQGCRPRF |
| PANMTAAVSLTRSPA |
| PANVLPFPVQRCPGC |
| PAPAPQRPLPRRPAL |
| PAPARASSSARSWRT |
| PAPCHLSHLWRQGPG |
| PAPDARRARVLWARS |
| PAPEARPRPGAAPQP |
| PAPFWPARPLLSAGI |
| PAPGEPWEAGGPHAG |
| PAPGPEWPGPRWWAP |
| PAPGQPGLGPGLSHP |
| PAPGQVQQPQLVPAP |
| PAPHHLCLLHAHLHH |
| PAPHPSPAACPWERA |
| PAPLPLVSSPRRRAS |
| PAPLQALLGRPPAPQ |
| PAPLQGLRDGGGWEA |
| PAPPPPLPAPPVQPL |
| PAPRSCPRPHTQARR |
| PAPSPFPALGALGQV |
| PASLPTATVRRCWTG |
| PASLVWHQTIQHPAQ |
| PASPASRPPSWLGFG |
| PASPGEVLPPRLFPL |
| PASPKGILHPTGPRL |
| PASPLHPSSCRASLI |
| PASRIPAPHGWPGLL |
| PASTFVTTGFGRSPL |
| PGGPAEGPSKRASPR |
| PGGPCRLPPPAPASA |
| PGGPGPVHLLLRHCW |
| PGGPLKPRWPACPGL |
| PGGPMWKWDLQIPFW |
| PGGPRATRVPQPRSE |
| PGGPSSGVVPLWVFL |
| PGPCAFHWAMGSTSP |
| PGPCCTRALSRRGMG |
| PGPDPLWENQVWGAV |
| PGPGGAPGHSGAGAA |
| PGPGGSCRIHGGGQL |
| PGPGQWLCPLHPQPG |
| PGPPEPAVCLQYSPE |
| PGPPLPPGLHVCWPA |
| PGPSAALTTMQLFSK |
| PGPSDESCTQASTSR |
| PGSPNAHSTAILSHK |
| PGSPWSCDHWSCGRC |
| PPAAPATVAMRPVEA |
| PPAAPCRRGAPSGAP |
| PPAAPGPATAAMLEV |
| PPAAPGPRAGPTGAE |
| PPAAPPPVTKARGCD |
| PPAAPWAWSCPSGVT |
| PPASPCACQPAAPAQ |
| PPGPAAALLQALGLR |
| PPGPAAEGGLYCPLG |
| PPGPPHSPQRRKCSP |
| PPGPQERARAARMMR |
| PPGPQPLGEGRELLL |
| PPGPRGHLRETACAL |
| PPGPRSASHCQPSLR |
| PPGPVHHALQRQSQR |
| PPGPWAHPRGSLLLY |
| PPPAFFSACPVCGGL |
| PPPAPAPAHGQQERG |
| PPPAPPASPLSLPTS |
| PPPAPTSSSSPSGGG |
| PPPGLASAAAAVRGA |
| PPPGPEGGLPSGNPW |
| PPPPPGWLPRLTRER |
| PPPSSPPQAAAAAIR |
| PPSNPRAWTPAGPPG |
| PPSPPCPDPAQVSSP |
| PRNPSAPPNQRGRHG |
| PRTADSISGPQGRSG |
| PSNLVSHHPLLGLLR |
| PSPGAPSSQGQAQPR |
| PSSPIPGPAEAHHDA |
| PSTDAAPGGVPAACA |
| PTPPPPAAPARSSPR |
| PTSPDWGPGAHRAAA |
| PTSPLPWPCPARGGA |
| PVPAKQHFRQWTRRC |
| PVPPVARTSPIPWLS |
| PVPRAQGAGASGCPS |
| PVPRGSPFAYTCPAA |
| PVPWLNSCHPTCPSA |
| PVSPATHRSGGRLLH |
| PVSPCQRHGGGRQHG |
| PVSPGALSYMESPTG |
| RPQLLAPAWNPASPG |
| SANPKSAFGHPLFSP |
| SARDLGWSEGCEDAR |
| SDSSAVSGCNPSCTP |
| SDSSNTEPWSMGHAR |
| SLKGASVKTGRRGGS |
| SPSVLSSSPSTSGAS |
| SSQPTASLSPSGPPS |
| SSRLPHLQQRLGCMF |
| SSSLLHMMMIPLHYY |
| SSSRLTTPQGRGAST |
| SSSRLWTATGTASVS |
| SSSVLTPTTPSGMRQ |
| STIPTVRSCSWEWPT |
| STLPMMFHLPPSVFM |
| STLPTQRSSGKRSSP |
| STSSRPRLNLSGMYG |
| SWSSHNSSFHHGNFY |
| SWSSRASSTPKAAWA |
| TSRDARPPGPKPTSS |
| TVNPPSPRAPPASSP |

**Additional file 1: Table S4. Classifying FSPs in response model without stable disease**

| AAAAAGAGGPGGGDA |
| --- |
| AERGAGAGGGRAAHQ |
| AGLAKVVEKSPGSCR |
| AIAWCWPPAAPTSGR |
| AILGKREKKEIQCSF |
| ANVPPGLLLGSLRAH |
| APAATAPRRAGAGRR |
| APAPAPDPAAPPARA |
| APAPRLQSARIPGRG |
| APLPFTPNRDRCSWQ |
| APLPSSWRPGTGTTI |
| APLPTPRLREVPHRF |
| APPPAGPAAAAAPRP |
| APPPALGTIFSCSPL |
| APPPCRPRVTPGPPP |
| APPPGPQLLWLCGLH |
| APPPPGGPGQAGDPA |
| APPPPPGFRCCAVSG |
| APPPVHTAPAGRGGP |
| APSPPRPRRRPTPTS |
| APVGAEGAAGSRLRP |
| APVPKSLHGGRVCLP |
| APVPPGPAACCCPHR |
| APVPRVCRDTATWFS |
| APVPSHQPVRRAAAQ |
| APVRTTAWASASIID |
| APVVPEASPRRALLP |
| ARRERRPRVSRLLTP |
| ASADAAGGEAAHDGH |
| ASADARPPWRSYRRC |
| GALLGSRAHAPWQPH |
| GALPRGPAVHQVPAA |
| GALPSADAGLAGGAA |
| GALQSASRLPSERRR |
| GAPAHPHRREALRLP |
| GAPALPLGHGDLPWQ |
| GAPAPPCSWTPPQQR |
| GAPAPSGEGRGTSHP |
| GAPLARQARQGLLLD |
| GAPLHMSPGTSHLSA |
| GAPLSLSPQRMHPLA |
| GAPLSPTSQSACTLA |
| GAPLVPAPHLHLWPH |
| GAPPEPATAAARSPD |
| GAPTEPSPTGRARGQ |
| GAPVASQRRRRRRRS |
| GAPVHPAWHHRDPGG |
| GAPVRRGCSQGPEQR |
| GAPVSGLPEGHAEAG |
| GGRGGGGGRGARLLG |
| GPPAVAPLVAGQPGL |
| GPPNALAQPRPPGWS |
| GPPPPAGRGDPARGG |
| GPTPLGCRCPAAPFS |
| GPVNILPPSWSSVPL |
| GPVPMRRVSELPGTL |
| GPVPQDLQEQQRRQV |
| IESVGSTSRTEHVGW |
| IKSVERLSLLSRHCH |
| ILTVDLHMDIWDMYS |
| ILTVSMTPCGAAGPH |
| LPGLPRGARPPQRAG |
| NGAPHLMGSGAVSSW |
| NPSAPSATVSPPCAV |
| NPSPGHCQMVQRPEW |
| NPSPHFRDFGHASSS |
| NPSPSCVQPSLPWLS |
| NSPPAAPSVPPAFQG |
| NSSPLAQSEVWRSWL |
| NTAPPTQSLGLAVHP |
| NVANLEKPLDLNKLY |
| NVSPSRPLPSLTTWA |
| PAAAAAAAPRSGRRL |
| PAAAAADHGLGRRFG |
| PAAAAVSGFCHQSLW |
| PAAAPARPWAERPRS |
| PAAAPLHQGRLAASD |
| PAAAPSSACRNSSST |
| PAAARCSSPGRPCAS |
| PAAGTWLLHGGSPSW |
| PAALHQRLHRLRAAQ |
| PAALPALRVAGCGGL |
| PAALPCPACHPTLTC |
| PAALPDAAAGGGARR |
| PAALPLRGHRPLRAP |
| PAALWTSGADRRVRE |
| PAARAGPAHHPQHPA |
| PAARALASAGPRHLQ |
| PAARIPGPPGPRGAE |
| PAARPAQLTAQLQRQ |
| PAARPPLLHDPRLLR |
| PAARQHRRPLLSGAV |
| PAARQQPGDPARGGG |
| PAARVPLLPPEPLPR |
| PAARVSPPWLRRSSA |
| PAAWENCLNARGSLP |
| PAAWRSGTGVAGGRC |
| PAAWTPPWETLSTWR |
| PAAYPPDCPLWLQGC |
| PAAYSSSSCLAAWAR |
| PAGNFHCGARRGESL |
| PAGPAGHAHAAQPGA |
| PAGPSCQILLLSPAT |
| PAGWSPSAQGCRPRF |
| PAHARCARAGCGCPR |
| PAHASRPHLHLRRDL |
| PAHPCPSWAPCPPGH |
| PAHPGSPPAAPACRR |
| PANMTAAVSLTRSPA |
| PANVLPFPVQRCPGC |
| PAPAPQRPLPRRPAL |
| PAPARASSSARSWRT |
| PAPCHLSHLWRQGPG |
| PAPDARRARVLWARS |
| PAPEARPRPGAAPQP |
| PAPFWPARPLLSAGI |
| PAPGEPWEAGGPHAG |
| PAPGICKSLRLPLLL |
| PAPGPEWPGPRWWAP |
| PAPGQPGLGPGLSHP |
| PAPGQVQQPQLVPAP |
| PAPGTYPGRAGSVGP |
| PAPHHLCLLHAHLHH |
| PAPHPSPAACPWERA |
| PAPLPLVSSPRRRAS |
| PAPLQALLGRPPAPQ |
| PAPLQGLRDGGGWEA |
| PAPPPPLPAPPVQPL |
| PAPRSCPRPHTQARR |
| PAPSLTTPTRARASC |
| PAPSPFPALGALGQV |
| PAPTGCRVSAGRQCP |
| PASASGTTWVFLSTS |
| PASECPGSPVAQPPF |
| PASLPTATVRRCWTG |
| PASLVWHQTIQHPAQ |
| PASPASRPPSWLGFG |
| PASPGEVLPPRLFPL |
| PASPKGILHPTGPRL |
| PASPLHPSSCRASLI |
| PASPQRAQLHPPGHV |
| PASRIPAPHGWPGLL |
| PASSALGVCPVPLPA |
| PASSPRWWTSRPKGI |
| PASTFVTTGFGRSPL |
| PASVPALEWPMTAVT |
| PGANLSQACCKYAIC |
| PGGPAEGPSKRASPR |
| PGGPAGPRGASPHPV |
| PGGPCRLPPPAPASA |
| PGGPGPVHLLLRHCW |
| PGGPLKPRWPACPGL |
| PGGPMWKWDLQIPFW |
| PGGPRATRVPQPRSE |
| PGPAGSGSIWTPYLP |
| PGPCAFHWAMGSTSP |
| PGPCCTRALSRRGMG |
| PGPDPLWENQVWGAV |
| PGPDQRLTAAPAWPQ |
| PGPGGAPGHSGAGAA |
| PGPGGSCRIHGGGQL |
| PGPGPVPSTDQSCGQ |
| PGPGQSTGPGGPGCR |
| PGPGQWLCPLHPQPG |
| PGPPEPAVCLQYSPE |
| PGPPLPPGLHVCWPA |
| PGPSAALTTMQLFSK |
| PGPSDESCTQASTSR |
| PGPSGPGWHSRSPAE |
| PGPTHQLPPHLPLLN |
| PGSPNAHSTAILSHK |
| PGSQASTESSAHPPT |
| PIPGPALSFPCPASL |
| PIPPAAALGQPPGDA |
| PPAAPATVAMRPVEA |
| PPAAPCRRGAPSGAP |
| PPAAPGPATAAMLEV |
| PPAAPGPRAGPTGAE |
| PPAAPPPVTKARGCD |
| PPAAPRRPRRPRQPR |
| PPAAPWAWSCPSGVT |
| PPAPAAWETHQNEVP |
| PPASPCACQPAAPAQ |
| PPGAPPAVWPGLLPH |
| PPGPAAALLQALGLR |
| PPGPAAEGGLYCPLG |
| PPGPGGRWHSPGDAA |
| PPGPPHSPQRRKCSP |
| PPGPQERARAARMMR |
| PPGPQERARAGRTMR |
| PPGPQPLGEGRELLL |
| PPGPRGHLRETACAL |
| PPGPRSASHCQPSLR |
| PPGPVHHALQRQSQR |
| PPGPWAHPRGSLLLY |
| PPGTPHHAPSGCHQP |
| PPPAFFSACPVCGGL |
| PPPAPAPAHGQQERG |
| PPPAPIPWSSPITNL |
| PPPAPPASPLSLPTS |
| PPPAPPPSQPVCTGL |
| PPPAPQECGLFCHHH |
| PPPAPTSSSSPSGGG |
| PPPDPTEPPAACQAG |
| PPPGHLLVPVLPAHL |
| PPPGLASAAAAVRGA |
| PPPGPEGGLPSGNPW |
| PPPLPLPPPYHWPGV |
| PPPPPGWLPRLTRER |
| PPPPPSQPGPSGTLP |
| PPPSPRPHPRRPPRS |
| PPPSSPPQAAAAAIR |
| PPPTPFDRHPQMSEP |
| PPSNPRAWTPAGPPG |
| PPSPDTLVVPHHQPH |
| PPSPPWWMTNLTTRR |
| PRNPSAPPNQRGRHG |
| PRTADSISGPQGRSG |
| PSNLVSHHPLLGLLR |
| PSPAHGPAACPACLS |
| PSPEQLLHRGRWLLA |
| PSPGAPSSQGQAQPR |
| PSPGPHNFPLGLHCQ |
| PSPPHAINNPPSFWA |
| PSSPIPGPAEAHHDA |
| PSSPLSPSPSAVSPT |
| PSSPSPHSGSLAQGG |
| PSSTCPPAAPTTLRA |
| PSTDAAPGGVPAACA |
| PTPPPPAAPARSSPR |
| PTSPDWGPGAHRAAA |
| PVPAAAGLWHGRGAL |
| PVPAKQHFRQWTRRC |
| PVPLPAGHPGRALPR |
| PVPPVARTSPIPWLS |
| PVPQPCCDFHFHRPF |
| PVPRAQGAGASGCPS |
| PVPRGSPFAYTCPAA |
| PVPVPCFSPAPGAPG |
| PVPWLNSCHPTCPSA |
| PVSPATHRSGGRLLH |
| PVSPCQRHGGGRQHG |
| PVSPGALSYMESPTG |
| RPPAAPAAPAAVPGP |
| RPQLLAPAWNPASPG |
| SANKRSTLRGKMKTS |
| SAPSRQSPAAEWRPR |
| SARAAATSACPVPRH |
| SARDLGWSEGCEDAR |
| SPSVLSSSPSTSGAS |
| SSQPTASLSPSGPPS |
| SSSLLHMMMIPLHYY |
| SSSRLISPTRRVGRR |
| SSSRLTTPQGRGAST |
| SSSRLWTATGTASVS |
| SSSVLTPTTPSGMRQ |
| SSVPSSPASRPKLPL |
| STIPTVRSCSWEWPT |
| STLPTQRSSGKRSSP |
| STVPRAEAVTKLGNC |
| TSRDARPPGPKPTSS |
| TSRPFTMSHGKEGMD |
| TSRPLVSPSRATGRR |
| TSRPRLPPPPRAPPL |
| TVNPPSPRAPPASSP |
| VPGPHSGRLNPASPC |

**Additional file 1: Table S5. Classifying FSPs in monotherapy response model**

SSSVLTPTTPSGMRQ

PPASPCACQPAAPAQ

APVPTGPRHAAPVRS

APVPGGVQNTWGPSG

GRSRSSVSLRAATGA

APVRTTAWASASIID

APVPPGPAACCCPHR

PPAAPCRRGAPSGAP

PNSPRCGYTVRRSSA

SSSRLISPTRRVGRR

PASPSAPRVPVGWSA

STSMTIRALSSTVSA

PASPHPWMALTSRPC

PPSPTVGSAPSQARV

PPSPESRLRGWRRNS

PPSPHRGFLLREDLD

PASPSWPGASVMTPW

APAPAPDPAAPPARA

PPSPNAAAKRSRRRT

PASPGAPLTLMGAFP

PGAPGRLCTRTPRRR

PPANSWPRKPPGKAL

PASPMACSPCTLGLA

PPAPCTGTATTSPST

PPAPWLSQPPSCHPS

PAPGSSCPSCSGYTL

PAPTASAAPTPQCPA

STILEDCPRGPEESG

PPSPVAPAPVIPTPG

NASPTSGSAMAALSA

PPSPCLQPLLQMSIL

PASPSSSWQSVGSSS

PPSPWRMGQSLRLKD

APVPRPQPQLRRGAA

GAPAPGLPARPGAAA

APVPKSLHGGRVCLP

APSPPRPRRRPTPTS

APPPAGPAAAAAPRP

GPAPPGTPGLPAPAA

GAPAPGAARPVSAEP

APPPPPGFRCCAVSG

PGAPLRLPPGLVLLL

PSSPIPGPAEAHHDP

SSSLLHMMMIPLHYY

PAPRTQRGVLPEQLC

SSSRLTTPQGRGAST

PGSPNAHSTAILSHK

PPPAPTSSSSPSGGG

PAGPRLHVPEAPVHA

PGPVATPPVRSPASR

PPSPSRRCCGPWWPD

PSPGSASPPWSWASW

PPSPTAAAPSAERCP

PAALDAPQAARLAGR

PAGPPWHPGPSRRSA

PAPRSCPRPHTQARR

PPSPPRGARPLPRVP

PAPSSSSPLWPGPTG

PPSPTIPCPTDSLLC

PAPSRASRVHGPPPE

PAPLLSYKDLVTKKS

PAPHPSPAACPWERA

PAARRCPRPARWVLC

PAGPALGPGHPAGAG

PASPVSPSTTWSLLG

PSSPQERQDHFQLWL

IRGNPAAPANGRGPS

STIPTVRSCSWEWPT

PSSPMWSTACRRTTW

PSSPLETETLRWRLM

PAPPASVPAAALGPG

PGSPSAHAELDRPPG

PPPSTAATTAAGASG

PSPAPPGPAGGQPRT

PAANGPESDGPHARA

PAGPRDRRAGRGRGA

PSSPPSMHLISGFGD

PPSPTRAGPTSRARR

PSSPRRIETTPVTSC

PGPPLPPGLHVCWPA

PSSPTTRWSSPSRAA

PASPATSSSPTARPG

PASPKWSWSLPGSPS

PTPPPPAAPARSSPR

PPSPRGRAPRCHDYS

PASPMEPLSPLPSPP

PVPLNCRAAPGVHLP

PPGPRFSALRTSQQV

PVPRTSSSPGFKLRN

PAARPHAWPLADHRL

PSSPGSAMASWHRAS

PASPGLLLCLQWAEE

PPSPWCHLGPGQQAR

PVPLASTGLAARGCA

NGPDLCGPLPGGCLG

GVTKPPSQPSAPSRS

PAPSPFPALGALGQV

PVSPCQRHGGGRQHG

PPGPTLRPPAEPAFL

PVSPGALSYMESPTG

PAARVPLLPPEPLPR

PSPTTAPACSPPPAW

PSPAGWGCSAGAGHV

PAPLLLHCPGLLQRD

PAGPAHPKPGCLPSL

PSPPPMSPANAPMPV

PAGPRGLAAARVGPL

PVSPGSSPTAAPGLV

PSSPACSAQGSSRAA

PPPAPPRPPSTAASS

PAAVPQMYPQGPARP

PASNKLPSLPLSKMY

PSSPAQHGPRPPGGR

CPPASPNPPAPLPRL

PSPAVAVSLSPPAEG

PGGPRATRVPQPRSE

PVPAKQHFRQWTRRC

PAGPRTPASRSASSS

PGANSRRSVNVQMAP

PAANPAATSPAAASP

PPSPPPSAISLPGPA

PPSPSWCCFSRDCWA

PAPQHCHPPGSGPSH

NPTAPSAAPERRCSS

PAGPAPSRLVHGAAL

PPSPGGAENGGKGPR

PPSPALLPASHQPCS

NPAPSLTSPWDGPGP

PASPPPSALSTAMGS

PAACPSGLCPKTPVG

PPSPGCCWRVCTSSS

PASPWRARLPAVPAR

PPSPWRRIPCWTQTC

PPPISPFSMTTMHTV

PPPGPLIAGSQNGGV

PAGPPPHCSQPGHLR

PSPAPWGSPSYCGAG

PPSPRCHLGPGHQAG

ASADAAGGEAAHDGH

PSPPVVGIAWVQAWE

PAAMGSWSLMNSKCS

PPGPAAAAFPRQGLQ

PGPDSDLHLRGHWRP

PAGPTTCLWFRRLSS

PAGPEALGLRQQRAA

PAPGPPHHPQHALLG

PPGPVHHALQRQSQR

SSQPTASLSPSGPPS

PASPPWRPSRTCCTD

PAPSSSLRPSPPPAA

PSSPCARTSARARPD

PPSPGSCRSWSAASK

PPSPCWAWTPGGWAA

PAAAPAALPAARSLF

PAAASLTRPHPEVLH

PGSPLGDTPPALCPE

AIAWCWPPAAPTSGR

PPGPQRPERRPAPSL

PPSPCRPARGSRIPA

PGSPPFSRSTSCRPD

PPSPLHPHPGEKWRE

PSPAPSLHPYRKAPK

PTSPSLLPYLEYPSL

PASPKGILHPTGPRL

PAPACGSVTDRNTDF

PANLEAIAKKGKFNK

PAPQLAWTLPSQRLS

STSILSAVSGVSSSP

PAGPADIRLDPGGPL

PPSPAPPPAPCPSRS

PAPCVSETLGQHDPA

PSPKPSPSPQLSLKF

PPSPFLGSLETTGAA

PPSPSSGELLLIQDP

PSPGQCPACGPCCAA

PGANLSHLSPLEAPP

PSSPTSTSTQGSPRR

PPSPSLAPAVPLAPR

PPPAFFSACPVCGGL

PGPGPGRPPGTAWPG

PSPTPAPTSCLGPSP

PAPLYDRMVHVRLLP

PGSPGKGAPLFPAVF

PVPLPSPRRRRSWRS

PVPRAQGAGASGCPS

PSSPRPARSHPRPAD

PGANGRSCRAFLPLL

PAPPRAPPAPGQPST

NPGAPLCTGQPTECH

PSANVCSPTTALDAA

PRNPSAPPNQRGRHG

PAPPAGRPVCTKQRP

PAAAAADHGLGRRFG

WNPTAPPSLTKQTGR

PASPDEYHAELVFLI

PPANSWPRKLPGKAP

PAGPVPSCGCEGAVD

PGSPLPPGPLHMHLL

PASPGEVLPPRLFPL

PAPGQPGLGPGLSHP

PVPWWQPPAPGPRWA

PGGPGPVHLLLRHCW

PVPRASPQWLLSTRT

PGPTASTAPAPTSSS

NPSPHFRDFGHASSS

PAASSPCPTGERSAS

PAPLSSAAPIPGSSL

PPPEPQQHHVNHRRA

PAAAASRCAGPSTVA

PGSPTGIHQTRSVSG

PAPLPLVSSPRRRAS

PPAAPGPATAAMLEV

PAGPRRPRPRGAHST

PAPLKLNFCSIPLAF

PSSPPCTPLTRLCRQ

PSNLVSHHPLLGLLR

PAAAPWSPRTTTRTR

PAGPAGEREPAGHLC

PAAAASCSFRLSATA

PGSPSTAVVTRVRGL

NGAPWTLLSGISSGT

PSSPPMPSSSSGRPR

PAPGQVQQPQLVPAP

PSPGPVCGCTMACTT

PSPGLSPLPASGLLS

PNSPVSSLSFPGLPF

PAPAHLHPVPVPAQR

PPSPAIECPSPPARL

PSPDATPSLQWTTAD

PASSSWDLRTLQTPQ

PPSPSSLLSPCNASI

PAAASALGAPVLPTA

PPSPSHHLPQPGGSA

PPPAPLGCARTSTPT

PPSPPTEAASSTARP

PAGPITCLCFRRLSS

PAAAPAAQRRLHPVL

PAGPPLGQHQPGRAA

PGSPRRCLWGPPGSM

PAGPVLPAPSSMPQT

PGANARHHRSTDSFV

STLPTQRSSGKRSSP

PAPQVCRAMTFCTRT

PPSPRSSPAPARAAV

PAPWLGGMLRCFSST

NTAPPTQSLGLAVHP

PGPAQLPRPGLPPRP

PSPSWQGSQGPLRAW

PSSPPRWPLPPSNHG

PAPPPGACLHLRLPE

PAAAPARPWAERPRS

PAARAPGLLPGHLGR

PAAWSSWRSWSTAWC

PGPSAALTTMQLFSK

PASPGAPGTCLHNPA

PSSPNRTLPPGRSGT

PAALPALRVAGCGGL

PAHADPLAGPTAWPS

PVPGRGCAAGVQAAG

PAPSSPPFTSNCVAS

PVPRKGPSQLAGQWQ

PAALPPAIPGHPVWT

PGSPCIWLGSPRSFL

PSPATSASLLLRNTQ

PTAPCSTHWACGCPC

PPAAPPPVTKARGCD

PAGPWQSHWPHRGLH

SPSVLSSSPSTSGAS

GPANPAAPPEEGSPP

PSSPIPGPAEAHHDA

PAPKTIPQQTHGTAP

PPAAPDAGMRPDDGG

PAGPRPVRSWRTCWR

PAARVSPPWLRRSSA

PAPGHQLHRLPHGPA

PAASPLPASGPSLEG

PAPSTVTRSTSPSLP

PGPPEPAVCLQYSPE

PPPAPPASPLSLPTS

PGSPSTATAASAAAD

PPSPHRAGPGLALWP

PPGPAAALLQALGLR

NPSPGHCQMVQRPEW

PASRPLDLVGVQHPL

PAASNTAQRLRRARV

PAGPASSGLVSPHAP

PAACSSRPSAGAPTA

PAPAAGHKFHLRHKQ

PAAEHLWNGRLLPNP

PASSPWSSCSPPWDC

PGPPPLHEAGPGPRS

GSCHNPAAPNHFQNL

PAGPAGHTRGTHKPF

PAPPPPLPAPPVQPL

PASPSPSSRLWLPSA

PAALPDAAAGGGARR

PPGPRGHLRETACAL

PPGPPHSPQRRKCSP

PAAMWRPGPTPRSGS

PAARIPAAAPGGLRA

PGPGQWLCPLHPQPG

PGSPEDNSALCRIPW

PAAYSSSSCLAAWAR

PASPSPTGWCTPTAL

PASPLHPSSCRASLI

PPGPSSSASSSQPGQ

PAACHRQALEATMRF

PASLPTATVRRCWTG

PAACTFPTKHRVRSA

PAPFDQPVLHQPLPL

PAPPAAHSGRGPHPV

PAGPSPTATAAPARR

PPPLPGLPGGAGKPG

PAPCPEPAGEPVPWE

PAGPPAAAALQQLRD

PTAPIQSSPRHGPQR

PGPDPLWENQVWGAV

PPPSSPPQAAAAAIR

PAGPGFQCGQGEDLK

PAPPHRGVRQIRLPA

PAHADGRRPRRGVCA

PPSNPRAWTPAGPPG

PAAYPPDCPLWLQGC

PPSPASSCWSWSTSS

PGPASCPPPPICGAL

PPSPESCTTARGPAA

PAGPTGPAGECSVPP

PAPACVWSSSPSSSC

PPSPLMSLWKMLLPF

PPSPPAATHGPSQRA

PAAAQQCSRGCPAGR

PPSPQKHLRICTSLG

PPAAPATVAMRPVEA

PGSPSRAHLWPTRRS

PGPWAEIAASSSGKT

PPSPSRRRAPPRSGR

PASPGARPSVPRRSR

PAPQMLSRWPTRRPG

PAPGPVLLGPSGFMG

PPPSLPPAWPGGGRG

PSSPDSTSRTRSPTR

PPSPMSTFPFNLEIH

PAPDARRARVLWARS

PPSPCSLPLPTMPGT

PAPGHHDGGRRAEAH

PSTDAAPGGVPAACA

PPSPGRLRLHLPAVD

PPSPAVPQAAQGASL

PGPGGAPGHSGAGAA

TSRDARPPGPKPTSS

NPAPPARACSSRWAE

PSPGAPSSQGQAQPR

PPSPAPSAPRAGPPG

PPSPTDHPGLHRLEI

PVPWLNSCHPTCPSA

PPSPAVSGDSQTPFC

PPSPAPRAQPSRARA

PPGPRSASHCQPSLR

PGGPAEGPSKRASPR

PGSPTRASSVAAAST

PAPGALPPAAPAGPA

PAPCGGRPAMTSVHL

PSPSPCAWCSPRGPS

PAGNFHCGARRGESL

PGSPALRPHPQSLPV

VTASWTNPASPSPCP

PAPPASFQNPATLLH

PPSPRSSTPTTCREP

PAHAWCPVLCTCPIV

PASCPCAHCPHASCQ

PAPGGYGSARTCRAL

PGPPASSTSMRSTRW

PAPTTTKSCRETFLK

PASNGRDRCQLDETA

PGGPLKPRWPACPGL

PAGPLCARSASAALF

PANWPAQPLCRHYSL

PAPGEPWEAGGPHAG

PASLVWHQTIQHPAQ

PEAPRLEFRDPALGL

PSSPGQANLYTYKPS

PSSPTRPAARPEAAV

PGPCCTRALSRRGMG

PGSPGSRWRCRRSRS

PASPTCSTVIWWPGW

PGAPGQGSARAAWGG

PGSPPSSCPNTGQEI

PASPASRPPSWLGFG

PVPPVARTSPIPWLS

PSSPSSRIFSQACLS

PPGPQERARAARMMR

PASCCPRAGCLRSPS

PANVLPFPVQRCPGC

PGPPGPELPHHGLCL

PPAAPWAWSCPSGVT

PAPPTAAPASGYRRG

PAGPCRHDLPEDIRK

PAGWSPSAQGCRPRF

PASRIPAPHGWPGLL

PAPLQGLRDGGGWEA

PPGPAPGAPAVPRLL

PGAPCPAPEPAPRLP

PAPLCAQAARAATLQ

PVPRGSPFAYTCPAA

PPPGPEGGLPSGNPW

PAPEARPRPGAAPQP

PVSPATHRSGGRLLH

PPGPGLRLALPGVQR

PVSPSPSTGLLLPAL

PASPRRLVPAGAAFL

PAAWPSARAPSRFPR

PVPRTSSGPGFKLRN

PPAAPGPRAGPTGAE

PAAACCPPGPPIPPA

PPAPWGSAAPAGQGL

NPSAPSATVSPPCAV

ASADARPPWRSYRRC

PVPRAPSSPCQEKGP

PGPPPAPAGPADPVV

NPAPTSFRPQQLPTA

PPSPVEPHSANPTSS

PAPAPRPAVLTVQRG

PAGPTSIHGWKTRAW

PANMTAAVSLTRSPA

PPSPVTRCPPTPGTM

PGGPCRLPPPAPASA

PAPCHLSHLWRQGPG

PPPPPAPRQLPPRPP

PPPAPAPAHGQQERG

PSPVTSSGVAPTSAA

PSPLPGCVIQHPLWP

PAAAPDRGGHPFRHG

PPPGLASAAAAVRGA

PASPPRRRRRPPPPR

PPSNRIRLHRLILPM

PSSPMSARSRRFSFP

PANSADFGTGAMAAG

PSPSRTSASFSTVVF

PGSPREPGVGGRCGG

PTPEPLAAKQVWGNH

PAPHPAAARSSPQPT

SARDLGWSEGCEDAR

PAANFLLKILSRLLI

PASPAAPYPRLRSRR

PPPGPGPGAASVLRA

PSPPQAAGVCGPSLP

PGNPAPQHCEAATPV

PSPCQALRPLSSYVP

PAPVPESAALPRAAA

PAGPGAAAAAAVRAV

PAPGPQLGRREDHAA

PSPPCATLQTATASW

PGPTAGTGDGTGHQP

PAGPGTPGPCSPSSS

PGPSPTSYPSPCWDP

NPSPSCVQPSLPWLS

SSSRLWTATGTASVS

NASPAPSASTATSAS

PAPLQALLGRPPAPQ

PSPSCLLTCGDSSAP

PGSNDSCASASRVAG

PAAAPSSACRNSSST

PGPSDESCTQASTSR

PAPATSTPDAAGSTL

PAPSQGSWPSARATW

PPGPVGCSRLGLRIR

PSAPWGPLYRAAPSQ

PPSPSRSSITASLAA

CPPAAPGPRPAVAAA

PAPPGAAVRATLRTG

PPPAPPPPSSPAAAA

PAPAPPPVGRPAGPA

PSPPPASTCQSQMLG

PSSPQLTWTGLMTWC

PASSTHGAGKSPSSL

PASTVWPPSLACAQR

PAPTAAAAAPAAGEE

PAPAPQRPLPRRPAL

PAPPMPHSLTISSVM

PAAHTGHHLYPPAPL

NPGAPLVGTPVDSHA

PSSPSWASAPCTWMR

RPQLLAPAWNPASPG

PSPVCPFPNTHNKTN

PSSPGNSSATTSGVW

PAPGPEWPGPRWWAP

PPSPPTWRPWGSLCL

PAPHHLCLLHAHLHH

NSSPSWSKGKRPGER

PAPQPTHLAHSAHSP

PAGPAHLHRSLGLHS

PAGPSCSACRRPPLA

PAPVPRLLATRSLPQ

PASTFVTTGFGRSPL

PAALHSLCGVLGGHA

PGGPLLTSTHPRAGA

PSSPSMRPSRTCCTH

PASPRIARYYDQCVW

APVPSHQPVRRAAAQ

PAAPVGAPCFRKPPS

SCLTEPRRDHKHRNV

GALPSADAGLAGGAA

APLPELVDKTRDTLP

APVLQKGGPEAEGGP

RTAPAAPRSRQPRPP

GAPAPPCSWTPPQQR

GPRGARRPRPPQSAA

APTPASVAPLPAAPL

GPPPPAGRGDPARGG

APPPCRPRVTPGPPP

GPPNALAQPRPPGWS

APLPTPRLREVPHRF

PRGARVRPRPRERRH

APPPPGGPGQAGDPA

APVPRVCRDTATWFS

APVPAYPEVWAIAWL

SARAAATSACPVPRH

VTQPNYGEKSHHHCL

ADPAVAPAAAPALSS

GAPLGAPGERAQAAF

GAPLSSAGRREPEQR

GAAPTASVSRPGVGG

EMDPQEYAFLICYQA

APAPRLQSARIPGRG

AILGKREKKEIQCSF

**Additional file 1: Table S6. Classifying FSPs in NSCLC response model**

PVPLPSPRRRRSWRS

PPGPQERARAARMMR

PAASSPCPTGERSAS

PASPASRPPSWLGFG

PGPPEPAVCLQYSPE

PAPGQVQQPQLVPAP

PAPPPPLPAPPVQPL

PGGPAEGPSKRASPR

PAPGQPGLGPGLSHP

PGGPLKPRWPACPGL

PPGPRSASHCQPSLR

PAALPALRVAGCGGL

PAACSSRPSAGAPTA

PPPAPAPAHGQQERG

SPSVLSSSPSTSGAS

PTPPPPAAPARSSPR

PANVLPFPVQRCPGC

SSSLLHMMMIPLHYY

PAPRSCPRPHTQARR

PASPLHPSSCRASLI

PASTFVTTGFGRSPL

PSPGAPSSQGQAQPR

STLPTQRSSGKRSSP

NPSAPSATVSPPCAV

PAPLQGLRDGGGWEA

SSQPTASLSPSGPPS

PAAAPSSACRNSSST

PAPGEPWEAGGPHAG

PPGPAAALLQALGLR

APVPPGPAACCCPHR

PAPLPLVSSPRRRAS

PGGPCRLPPPAPASA

PAAYSSSSCLAAWAR

PVSPCQRHGGGRQHG

PAPEARPRPGAAPQP

PPPGLASAAAAVRGA

PGPSDESCTQASTSR

PASLPTATVRRCWTG

RPQLLAPAWNPASPG

PAAYPPDCPLWLQGC

PAAAAADHGLGRRFG

NPSPGHCQMVQRPEW

PPPAPPASPLSLPTS

PRNPSAPPNQRGRHG

PSSPIPGPAEAHHDA

PPAAPATVAMRPVEA

NPSPHFRDFGHASSS

PAAAQQCSRGCPAGR

PGPCCTRALSRRGMG

PPGPPHSPQRRKCSP

SSSRLWTATGTASVS

PAPGPEWPGPRWWAP

PVSPGALSYMESPTG

PASPKGILHPTGPRL

PGPPLPPGLHVCWPA

PAPHPSPAACPWERA

PAPAPQRPLPRRPAL

APAPAPDPAAPPARA

PVPRGSPFAYTCPAA

PAGNFHCGARRGESL

PSTDAAPGGVPAACA

PGGPGPVHLLLRHCW

PAPLQALLGRPPAPQ

PPPSSPPQAAAAAIR

PAAAPARPWAERPRS

PVPPVARTSPIPWLS

NPSPSCVQPSLPWLS

PPGPVHHALQRQSQR

PGGPRATRVPQPRSE

PGPSAALTTMQLFSK

PPAAPWAWSCPSGVT

AIAWCWPPAAPTSGR

PPASPCACQPAAPAQ

PPAAPGPATAAMLEV

PVPRAQGAGASGCPS

PPAAPPPVTKARGCD

PANMTAAVSLTRSPA

PPAAPCRRGAPSGAP

NTAPPTQSLGLAVHP

PAGWSPSAQGCRPRF

PAPSPFPALGALGQV

PGSPNAHSTAILSHK

PGPDPLWENQVWGAV

PPAAPGPRAGPTGAE

PAPHHLCLLHAHLHH

TSRDARPPGPKPTSS

PAARVPLLPPEPLPR

PAPCHLSHLWRQGPG

PAACTFPTKHRVRSA

PASPGEVLPPRLFPL

PPGPRGHLRETACAL

PAAASLTRPHPEVLH

PAARVSPPWLRRSSA

PASLVWHQTIQHPAQ

PSNLVSHHPLLGLLR

PPPAFFSACPVCGGL

PVSPATHRSGGRLLH

PGPGGAPGHSGAGAA

PAPDARRARVLWARS

PPPGPEGGLPSGNPW

PPPAPTSSSSPSGGG

PAALPDAAAGGGARR

PPSNPRAWTPAGPPG

PVPWLNSCHPTCPSA

PGPGQWLCPLHPQPG

PVPAKQHFRQWTRRC

SSSRLTTPQGRGAST

PASRIPAPHGWPGLL

APVPKSLHGGRVCLP

APPPAGPAAAAAPRP

PAPFWPARPLLSAGI

SSSVLTPTTPSGMRQ

APPPPPGFRCCAVSG

AILGKREKKEIQCSF

PAALPLRGHRPLRAP

VDLVLEAPDEEHRPQ

NSSPLAQSEVWRSWL

RPPAAPAAPAAVPGP

PAAAAAAAPRSGRRL

PSPGPHNFPLGLHCQ

PAALPCPACHPTLTC

PPPAPQECGLFCHHH

PGPGQSTGPGGPGCR

PAAAAVSGFCHQSLW

PAASSPTSCLMDPRP

PGPDQRLTAAPAWPQ

PVPAAAGLWHGRGAL

PAPSLTTPTRARASC

PAPGICKSLRLPLLL

PAAGTWLLHGGSPSW

PPGPAAEGGLYCPLG

PPGTPHHAPSGCHQP

PAHARCARAGCGCPR

PAARPAQLTAQLQRQ

PAARAGPAHHPQHPA

PSPEQLLHRGRWLLA

PAHPCPSWAPCPPGH

PSPPHAINNPPSFWA

PAAETLPTRSWETHV

PAPTGCRVSAGRQCP

PAALHQRLHRLRAAQ

PGPCAFHWAMGSTSP

PGSQASTESSAHPPT

VDTARNLPPSMRRPP

PGGPAGPRGASPHPV

PASSPRWWTSRPKGI

PASASGTTWVFLSTS

PVPVPRPPAPLSRAP

PGPTHQLPPHLPLLN

PAARIPGPPGPRGAE

PAARQHRRPLLSGAV

PSSTCPPAAPTTLRA

PAARPPLLHDPRLLR

PASVPALEWPMTAVT

PVPQPCCDFHFHRPF

PPPAPIPWSSPITNL

PPPDPTEPPAACQAG

PPPAPPPSQPVCTGL

PPPSPRPHPRRPPRS

PSSPLSPSPSAVSPT

VPGPHSGRLNPASPC

PPSPDTLVVPHHQPH

PASECPGSPVAQPPF

PPGPQERARAGRTMR

PAAWRSGTGVAGGRC

PAARALASAGPRHLQ

PGPSGPGWHSRSPAE

PPGPGGRWHSPGDAA

PAALWTSGADRRVRE

PPPTPFDRHPQMSEP

PAHASRPHLHLRRDL

PGPGGSCRIHGGGQL

PAHPGSPPAAPACRR

PAPGTYPGRAGSVGP

PGPAGSGSIWTPYLP

PPGAPPAVWPGLLPH

NVSPSRPLPSLTTWA

PIPPAAALGQPPGDA

ASADARPPWRSYRRC

PGANLSQACCKYAIC

PPPLPLPPPYHWPGV

SARDLGWSEGCEDAR

PPPPPSQPGPSGTLP

STIPTVRSCSWEWPT

PAAWTPPWETLSTWR

ASADAAGGEAAHDGH

PAARAPPPPRAPPPP

GAPVSGLPEGHAEAG

PAAARCSSPGRPCAS

PPSPPWWMTNLTTRR

PGPGPVPSTDQSCGQ

PAARQQPGDPARGGG

PSPAHGPAACPACLS

PPPGHLLVPVLPAHL

PASSALGVCPVPLPA

PSSPSPHSGSLAQGG

APSPPRPRRRPTPTS

APPPCRPRVTPGPPP

GAPAPPCSWTPPQQR

GPRGARRPRPPQSAA

GPPPPAGRGDPARGG

PPGPWAHPRGSLLLY

PAGPAGHAHAAQPGA

PAGPIPCAEGLHAVP

PAAAPLHQGRLAASD

NGAPHLMGSGAVSSW

PPGPQPLGEGRELLL

PAPARASSSARSWRT

PPPPPGWLPRLTRER

PTSPDWGPGAHRAAA

AAAAAGAGGPGGGDA

APVPSHQPVRRAAAQ

PGGPMWKWDLQIPFW

NVANLEKPLDLNKLY

APVPRVCRDTATWFS

PAAAAALPPHGAGAD

GALPSADAGLAGGAA

TVNPPSPRAPPASSP

PAAWENCLNARGSLP

PAGPSCQILLLSPAT

NSPPAAPSVPPAFQG

APPPPGGPGQAGDPA

APLPTPRLREVPHRF

STSMTIRALSSTVSA

VSVRSTGKQPRTVTP

VAPRAARGRRAPGQG

PRGARVRPRPRERRH

PRTADSISGPQGRSG

PPAAPRRPRRPRQPR

VTASWTNPASPSPCP

SGDFSLLPDPSGTTL

SIVDFTSAFLSGCHT

SSSLLCWRGGVCRMP

KKGLPAPSVLLSWRW

STLPLWQPPGLPSRD

VDTLRHVINQTGGYS

VDTRVHTLVGLVWKV

VDTPAWVLHKAYRCG

VDLVPGRVHRVWQLP

VDLPAGRARRPRADR

SARAAATSACPVPRH

VDLQEHALAAAAGHF

SAPPPLRPQPRPLPQ

ADSSGPFRPHVQFIC

SDSSNTEPWSMGHAR

FSSSAPESEISTTIP

IMTVASEKPHNKLLK

ISTVAHSDSSKTSPE

PVPQHRPGAPRDEPL

PVPGPWADGRWSVPR

PPGPIRVLPSGDRLF

PEAPTASKLYKQYGG

PVPQGFTRCPRGGPS

PPGPLMSLWKMLLPL

PAAVLPGSEFPGQHL

AAPLLPSTFHTFPVG

PAPLRGSLVNVIHPG

PAALGRLEPPGFPAP

VLVPAAPAGHTGGPQ

PAASAAWGPSPSAAS

PASSRSWPPFRIKAG

PAACGRPPAARPRPW

PHAPTGRARLGVRGR

PPSNPGGAPEVHQLL

AAPSAGCFGTSYFRI

PAAKSAKSFPRRLRD

FRLHVKKETKDASTI

PAATWPIATSAIAPL

WPPAAPRSSGAACCS

PAASQCFSAPAGSCP

PAAALQTQHCARVPH

PAAARSPAGGHAHPV

PAALAQGLGAAHLGL

PAARFCSDSPPQELL

PPAAAGYHAYWKPAA

PAALPLLAGRCTREV

PASASWPCPSAAWRC

PAAGSSLCLGLLGSL

PAPWSPRACAKPAGV

PAARGQGLQCAPLHF

WPPPPPAPVSPTTTS

**Additional file 1: S7. Classifying FSPs in adverse event model**

| \| EMPPLKAPRAKVPKA \| \| --- \| \| GGAASSLDSLKLASR \| \| GSLSSLESATTDSDL \| \| LESRARRSTCATSPT \| \| PGAGAGVYVHGQAEP \| \| QLVPALGPPVRAELL \| \| SGAGAGWLQPGREPP \| \| SLATHLIWTQRQWMM \| \| SLCTMLSPSTRPRSL \| \| SLFTHVKASRIPAEF \| \| SLQTHLLRPEVRVDC \| \| SPCSPPRGGRLAEGP  WPVEARVGCREDRWD \| |
| --- | --- | --- | --- | --- | --- | --- | --- | --- | --- | --- | --- | --- |

**Additional file 1: Table S8. GO analysis of source genes for the classifying FSPs comprising the response without SD and monotherapy response models.**

| **PANTHER Overrepresentation Test (Released 20221013)** | | | | | | | |
| --- | --- | --- | --- | --- | --- | --- | --- |
| **GO Ontology database DOI: 10.5281/zenodo.6799722 Released 2022-07-01** | | | | | | | |
| **Homo sapiens (all genes in database)** | | | | | | | |
| **Test Type: FISHER** | | | | | | | |
| **Correction: FDR** | | | | | | | |
|  | | | | | | | |
| **Analyzed List: 266 FSP from response without SD model** | | | | | | | |
| **GO cellular component complete** | Homo sapiens - REFLIST (20589) | 266 FSP (266) | 266 FSP (expected) | 266 FSP (over/ under) | 266 FSP (fold Enrich-ment) | 266 FSP (raw *p*-value) | 266 FSP (FDR) |
| postsynaptic density (GO:0014069) | 326 | 17 | 4.21 | + | 4.04 | 2.18E-06 | 0.00445 |
| asymmetric synapse (GO:0032279) | 332 | 17 | 4.29 | + | 3.96 | 2.75E-06 | 0.00187 |
| postsynaptic specialization (GO:0099572) | 347 | 17 | 4.48 | + | 3.79 | 4.83E-06 | 0.00197 |
| neuron to neuron synapse (GO:0098984) | 357 | 17 | 4.61 | + | 3.69 | 6.9E-06 | 0.00235 |
| postsynapse (GO:0098794) | 626 | 22 | 8.09 | + | 2.72 | 3.22E-05 | 0.0094 |
| anchoring junction (GO:0070161) | 1336 | 39 | 17.26 | + | 2.26 | 2.46E-06 | 0.00251 |
| synapse (GO:0045202) | 1360 | 35 | 17.57 | + | 1.99 | 0.000161 | 0.0365 |
| cell junction (GO:0030054) | 2136 | 53 | 27.6 | + | 1.92 | 4.27E-06 | 0.00218 |
| cell periphery (GO:0071944) | 6460 | 113 | 83.46 | + | 1.35 | 0.000187 | 0.0381 |
| membrane (GO:0016020) | 9956 | 163 | 128.63 | + | 1.27 | 3.23E-05 | 0.00825 |
|  | | | | | | | |
| **GO molecular function complete** | Homo sapiens - REFLIST (20589) | 266 FSP (266) | 266 FSP (expected) | 266 FSP (over/under) | 266 FSP (fold Enrichment) | 266 FSP (raw P-value) | 266 FSP (FDR) |
| guanyl-nucleotide exchange factor activity (GO:0005085) | 228 | 14 | 2.95 | + | 4.75 | 2.98E-06 | 0.0148 |
| GTPase regulator activity (GO:0030695) | 494 | 20 | 6.38 | + | 3.13 | 1.08E-05 | 0.027 |
| nucleoside-triphosphatase regulator activity (GO:0060589) | 494 | 20 | 6.38 | + | 3.13 | 1.08E-05 | 0.018 |
|  | | | | | | | |
| **Analyzed List: 525 FSP from monotherapy response models** | | | | | | | |
| **GO molecular function complete** | Homo sapiens - REFLIST (20589) | 525 FSP (496) | 525 FSP (expected) | 525 FSP (over/ under) | 525 FSP (fold Enrich-ment) | 525 FSP (raw *p*-value) | 525 FSP (FDR) |
| molecular_function (GO:0003674) | 18292 | 469 | 440.66 | + | 1.06 | 1.67E-05 | 0.0829 |
| Unclassified (UNCLASSIFIED) | 2297 | 27 | 55.34 | - | 0.49 | 1.67E-05 | 0.0415 |
|  | | | | | | | |
| **GO biological process complete** | Homo sapiens - REFLIST (20589) | 525 FSP (496) | 525 FSP (expected) | 525 FSP (over/under) | 525 FSP (fold Enrichment) | 525 FSP (raw P-value) | 525 FSP (FDR) |
| detection of stimulus involved in sensory perception (GO:0050906) | 560 | 0 | 13.49 | - | < 0.01 | 2.81E-06 | 0.0441 |
|  | | | | | | | |
| **GO cellular component complete** | Homo sapiens - REFLIST (20589) | 525 FSP (496) | 525 FSP (expected) | 525 FSP (over/under) | 525 FSP (fold Enrichment) | 525 FSP (raw P-value) | 525 FSP (FDR) |
| plasma membrane region (GO:0098590) | 1257 | 54 | 30.28 | + | 1.78 | 6.49E-05 | 0.0662 |
| cell projection (GO:0042995) | 2391 | 90 | 57.6 | + | 1.56 | 2.83E-05 | 0.0577 |
| plasma membrane bounded cell projection (GO:0120025) | 2282 | 85 | 54.97 | + | 1.55 | 6.89E-05 | 0.0469 |

**Additional file 1: Table S9. Expression levels* of source genes for irAE-classifying FSPs.**

| **Tissue** | **DNMT1** | **NVL** | **RABGEF1** | **SEC14L4** | **SEPTIN4** | **ATP8B3** | **ECT2L** |
| --- | --- | --- | --- | --- | --- | --- | --- |
| **adipocyte** | N/A | N/A | 3.944 | N/A | 3.635 | 4.301 | N/A |
| **adipose tissue** | 3.369 | N/A | N/A | N/A | 5.066 | N/A | N/A |
| **adrenal gland** | 3.605 | 4.431 | 3.406 | 4.736 | 5.130 | 3.993 | N/A |
| **blood platelet** | 4.336 | N/A | 3.757 | N/A | N/A | N/A | N/A |
| **B-lymphocyte** | 4.110 | 4.286 | 5.074 | N/A | N/A | N/A | N/A |
| **bone marrow** | 5.786 | 5.042 | N/A | N/A | N/A | N/A | N/A |
| **brain** | 3.745 | 4.368 | 5.210 | N/A | 6.088 | 4.453 | N/A |
| **breast** | 4.575 | N/A | N/A | N/A | N/A | N/A | N/A |
| **breast cancer cell** | 4.835 | 4.907 | N/A | N/A | 3.800 | 3.205 | N/A |
| **carotid atherosclerotic plaque** | N/A | N/A | N/A | N/A | 4.166 | N/A | N/A |
| **cerebral cortex** | N/A | N/A | N/A | N/A | N/A | 3.814 | N/A |
| **cervical mucosa** | N/A | N/A | N/A | N/A | 5.521 | N/A | N/A |
| **colon** | 4.103 | 3.282 | 3.599 | N/A | 4.723 | 4.339 | 5.182 |
| **cytotoxic T-lymphocyte** | 4.984 | 4.659 | 4.714 | N/A | N/A | 3.778 | N/A |
| **duodenum** | 4.465 | 5.044 | N/A | N/A | 4.576 | N/A | 2.678 |
| **embryonic stem cell** | 5.386 | 5.279 | N/A | N/A | N/A | N/A | N/A |
| **esophagus** | 3.940 | 5.418 | N/A | N/A | 4.379 | N/A | N/A |
| **gall bladder** | 4.127 | 3.051 | 4.073 | 4.606 | 4.681 | 3.746 | N/A |
| **gut** | 4.358 | N/A | 4.009 | N/A | N/A | 4.457 | 3.739 |
| **heart** | 3.925 | N/A | 3.812 | N/A | 3.549 | 3.594 | N/A |
| **helper T-lymphocyte** | 4.431 | 3.614 | 3.884 | N/A | N/A | N/A | N/A |
| **kidney** | 3.555 | 2.649 | N/A | N/A | 4.692 | N/A | N/A |
| **liver** | 4.000 | 3.676 | 3.647 | 4.621 | 4.964 | N/A | 4.190 |
| **lung** | 3.709 | 4.736 | 3.400 | 4.010 | 4.360 | 3.850 | 4.759 |
| **lymph node** | 5.171 | 4.494 | 4.328 | N/A | 4.369 | N/A | N/A |
| **mesenchymal stem cell** | 4.839 | N/A | N/A | N/A | N/A | N/A | N/A |
| **monocyte** | 4.317 | 3.852 | 4.145 | N/A | N/A | N/A | N/A |
| **myometrium** | 3.393 | N/A | 3.548 | N/A | N/A | 4.430 | 4.495 |
| **natural killer cell** | 4.911 | 3.123 | 4.447 | N/A | N/A | 3.164 | N/A |
| **osteosarcoma cell** | 3.652 | N/A | N/A | N/A | N/A | N/A | N/A |
| **ovary** | 3.809 | 4.123 | 4.187 | 4.561 | 4.551 | 3.916 | N/A |
| **oviduct** | 5.003 | 4.318 | N/A | 3.789 | 4.135 | N/A | 4.742 |
| **pancreas** | 3.783 | 3.442 | 3.955 | N/A | 4.965 | N/A | N/A |
| **pancreatic islet** | 2.815 | N/A | 3.215 | N/A | N/A | N/A | 3.207 |
| **placenta** | 4.451 | 4.568 | 4.232 | 4.565 | 4.480 | 4.126 | N/A |
| **prefrontal cortex** | 4.386 | N/A | 4.104 | N/A | 5.853 | 3.853 | N/A |
| **prostate gland** | 3.726 | 4.186 | 3.877 | N/A | 3.874 | N/A | N/A |
| **rectum** | 4.166 | 3.643 | 3.588 | 3.069 | 4.092 | 4.512 | N/A |
| **retina** | 3.654 | 3.309 | 4.414 | N/A | 5.820 | 4.902 | N/A |
| **salivary gland** | 3.793 | 3.624 | N/A | 3.881 | 4.327 | N/A | N/A |
| **seminal vesicle** | N/A | N/A | 3.642 | N/A | N/A | 3.037 | N/A |
| **small intestine** | 4.425 | 4.620 | N/A | N/A | 4.496 | N/A | N/A |
| **smooth muscle** | 5.203 | 3.044 | N/A | N/A | 4.348 | N/A | N/A |
| **spermatozoon** | 3.111 | N/A | N/A | N/A | 5.446 | 4.403 | N/A |
| **spinal cord** | 4.637 | N/A | 4.109 | N/A | 5.662 | N/A | N/A |
| **spleen** | 4.221 | 2.985 | 4.294 | 4.567 | 5.621 | N/A | N/A |
| **stomach** | 4.423 | 3.357 | 4.075 | N/A | 4.065 | N/A | N/A |
| **testis** | 4.026 | 4.057 | 4.337 | 4.801 | 4.155 | N/A | N/A |
| **thyroid gland** | 4.593 | 4.101 | N/A | N/A | 4.356 | N/A | N/A |
| **tonsil** | 5.226 | 4.350 | N/A | N/A | 4.327 | N/A | 3.519 |
| **urinary bladder** | 4.137 | 3.940 | 4.342 | 3.753 | 3.996 | 4.135 | N/A |
| **uterine cervix** | 2.492 | N/A | N/A | N/A | N/A | N/A | N/A |
| **uterine endometrium** | 5.080 | 4.016 | N/A | N/A | 4.366 | 3.844 | N/A |
| **uterus** | 4.517 | N/A | 3.364 | N/A | N/A | N/A | N/A |
| **vermiform appendix** | 4.955 | 4.174 | N/A | N/A | 3.739 | N/A | N/A |

* The expression level of each protein was determined from [www.proteomicsdb.org](http://www.proteomicsdb.org). It is a MComBat normalized value for each protein in different tissues. Total sum normalized proteomic expression values were calculated using their database as a reference set.
